# Iron Regulation in *Clostridioides difficile*

**DOI:** 10.3389/fmicb.2018.03183

**Published:** 2018-12-24

**Authors:** Mareike Berges, Annika-Marisa Michel, Christian Lassek, Aaron M. Nuss, Michael Beckstette, Petra Dersch, Katharina Riedel, Susanne Sievers, Dörte Becher, Andreas Otto, Sandra Maaß, Manfred Rohde, Denitsa Eckweiler, Jose M. Borrero-de Acuña, Martina Jahn, Meina Neumann-Schaal, Dieter Jahn

**Affiliations:** ^1^Braunschweig Integrated Centre of Systems Biology (BRICS), Technische Universität Braunschweig, Braunschweig, Germany; ^2^Center for Functional Genomics of Microbes (CFGM), Institute of Microbiology, University of Greifswald, Greifswald, Germany; ^3^Department of Molecular Infection Biology, Helmholtz Centre for Infection Research (HZI), Braunschweig, Germany; ^4^Central Facility for Microscopy, Helmholtz Centre for Infection Research (HZI), Braunschweig, Germany; ^5^Institute of Microbiology, Technische Universität Braunschweig, Braunschweig, Germany; ^6^Leibniz-Institute DSMZ–German Collection of Microorganisms and Cell Cultures, Braunschweig, Germany

**Keywords:** Fur, iron regulation, metabolism, iron transport, cell wall, polyamine

## Abstract

The response to iron limitation of several bacteria is regulated by the ferric uptake regulator (Fur). The Fur-regulated transcriptional, translational and metabolic networks of the Gram-positive, pathogen *Clostridioides difficile* were investigated by a combined RNA sequencing, proteomic, metabolomic and electron microscopy approach. At high iron conditions (15 μM) the *C. difficile fur* mutant displayed a growth deficiency compared to wild type *C. difficile* cells. Several iron and siderophore transporter genes were induced by Fur during low iron (0.2 μM) conditions. The major adaptation to low iron conditions was observed for the central energy metabolism. Most ferredoxin-dependent amino acid fermentations were significantly down regulated (*had, etf, acd, grd, trx, bdc, hbd*). The substrates of these pathways phenylalanine, leucine, glycine and some intermediates (phenylpyruvate, 2-oxo-isocaproate, 3-hydroxy-butyryl-CoA, crotonyl-CoA) accumulated, while end products like isocaproate and butyrate were found reduced. Flavodoxin (*fldX*) formation and riboflavin biosynthesis (*rib*) were enhanced, most likely to replace the missing ferredoxins. Proline reductase (*prd*), the corresponding ion pumping RNF complex (*rnf*) and the reaction product 5-aminovalerate were significantly enhanced. An ATP forming ATPase (*atpCDGAHFEB*) of the F_0_F_1_-type was induced while the formation of a ATP-consuming, proton-pumping V-type ATPase (*atpDBAFCEKI*) was decreased. The [Fe-S] enzyme-dependent pyruvate formate lyase (*pfl*), formate dehydrogenase (*fdh*) and hydrogenase (*hyd*) branch of glucose utilization and glycogen biosynthesis (glg) were significantly reduced, leading to an accumulation of glucose and pyruvate. The formation of [Fe-S] enzyme carbon monoxide dehydrogenase (*coo*) was inhibited. The *fur* mutant showed an increased sensitivity to vancomycin and polymyxin B. An intensive remodeling of the cell wall was observed, Polyamine biosynthesis (*spe*) was induced leading to an accumulation of spermine, spermidine, and putrescine. The *fur* mutant lost most of its flagella and motility. Finally, the CRISPR/Cas and a prophage encoding operon were downregulated. Fur binding sites were found upstream of around 20 of the regulated genes. Overall, adaptation to low iron conditions in *C. difficile* focused on an increase of iron import, a significant replacement of iron requiring metabolic pathways and the restructuring of the cell surface for protection during the complex adaptation phase and was only partly directly regulated by Fur.

## Introduction

*Clostridioides difficile* (formerly *Clostridium difficile*) is a spore-forming, Gram-positive, anaerobic, toxins-producing pathogen leading to often hospital-acquired infections worldwide (Burke and Lamont, 2014). The phenotypes of *C. difficile* infections (CDI) range from mild diarrhea to toxic megacolon which ultimately causes death (Bartlett and Gerding, 2008). In the United States over half a million cases of CDI per year with approximately 30,000 deaths are reported, making CDI to one of the most common and also cost-effective healthcare-associated infections (Lessa et al., 2015). Proteins containing iron, [Fe-S]-clusters and iron-coordinated heme are indispensable for the bacterial metabolism. Consequently, iron is an essential element for the growth of all bacteria including *C. difficile* (Symeonidis, 2012). Despite its abundance in nature, iron is often a growth-limiting nutrient due to the low solubility of the dominating oxidized ferric iron over the soluble ferric form (Braun and Hantke, 2011). To counteract this problem, bacteria have developed high affinity transporters and high affinity chelators, so called siderophores, which are excreted and re-imported after iron acquisition to cope with this limitation (Huang and Wilks, 2017; Khan et al., 2018). Alternatively, ferric reductases are excreted (Schroder et al., 2003). In pathogenic bacteria these iron-uptake mechanisms acquire iron directly from host proteins, including the iron-binding glycoproteins transferrin in serum and extracellular fluid, lactoferrin in mucosal secretions, and heme-containing proteins such as hemoglobin, haptoglobin, and hemopexin (Symeonidis, 2012). *C. difficile* can utilize different iron salts (FeCl_3_, FeSO_4_), iron citrate and ferritin as iron source (Cernat and Scott, 2012). In a previous investigation ferritin, hemoproteins and heme were able to sustain growth of *C. difficile* under iron-limited condition (Cernat and Scott, 2012). However, a cellular overload with iron has to be avoided to prevented reactive oxygen species generation via the Fenton reaction (Cornelis et al., 2011). As a consequence, bacteria have evolved various mechanisms to control iron homeostasis. They carefully adjust their iron uptake and utilization strategies at the transcriptional level (Troxell and Hassan, 2013; Porcheron and Dozois, 2015). Several iron-responsive regulatory proteins (Fur, Irr, RirA, and IscR) have been described in bacteria (Rudolph et al., 2006; Santos et al., 2015; Mandin et al., 2016). The ferric uptake regulator (Fur) protein is a transcriptional repressor of genes in iron uptake and utilization (Troxell and Hassan, 2013; Fillat, 2014; Porcheron and Dozois, 2015). The Fur protein typically contains two structural domains, the N-terminal DNA binding domain and the C-terminal dimerization domain (Deng et al., 2015). Under iron-replete conditions, Fe^2+^ functions as a co-repressor in that the Fur–Fe^2+^ complex binds a conserved DNA site in the promoter of a regulated gene and usually inhibits the expression. In contrast, under iron starvation conditions, the Fur protein is inactive, which allows for the expression of Fur-regulated genes.

The Fur regulons of *Clostridium acetobutylicum* and *C. difficile* were determined using DNA microarray-based transcriptome analyses (Vasileva et al., 2012; Ho and Ellermeier, 2015). In *C. difficile* one transcriptome investigation focused on high iron versus iron-depleted conditions (Hastie et al., 2018), while the second defined the Fur-regulon under high iron conditions (Ho and Ellermeier, 2015). In *C. acetobutylicum* genes for various siderophore uptake systems (*feo*, *fhu*), a flavodoxin (*fldX*), lactate dehydrogenase (*ldh*), benzoyl-CoA reductase and riboflavin biosynthesis (*rib*) were found under Fur-mediated iron control. A Fur binding site of G/T-A/T-T/G-A-A-T-N-A/T-T/A-T/A- T-C-A-T/A-T/A-A/T was proposed (Vasileva et al., 2012). Similarly, in *C. difficile* genes for 7 putative cation transport systems including various iron uptake systems (*fpi*, *feo*, and *fhu*,) a flavodoxin (*fldX*), two component regulatory systems and very few metabolic enzymes were found repressed by Fur in an iron-dependent manner. But also a series of Fur induced genes were identified. Furthermore, *in vitro* DNA binding by Fur was shown (Ho and Ellermeier, 2015). The deduced Fur binding site was A-A-A-T-G-A-T-A-A-T-N-A-A/T-T/A-A/T-T-C-A. A similar binding site A/T-A/T-N- T/A-N-T-G-A-T-A-A-T-G-A-T-T-T-T-C-A-T-T-A/T was proposed by (Dubois et al., 2016). They demonstrated cysteine-dependent regulation of *fur* and several *fur* target genes. Finally, the *C. difficile* Fur regulon was found induced in a hamster infection model control (Ho and Ellermeier, 2015). During the second DNA-array-based transcriptome approach focusing on iron versus iron-depleted conditions, genes for a flavodoxin, enzymes of polyamine and histidine biosynthesis, and flagella formation were found induced under iron limiting conditions (Hastie et al., 2018). Corresponding studies in *Clostridium perfringens* identified FeoB as the major systems to counteract iron depletion in this bacterium (Awad et al., 2016). Finally, a bioinformatics investigation proposed the DNA binding site for *Clostridium botulinum* Fur as A/T-T/A- T-N-A/T-T/A- A-A/T-T-A/T-A-T/A-T/A-A-T-T-A/T-T-T (Zhang et al., 2011). A position weight matrix analyses was employed for regulon prediction.

Here we describe a combined RNA sequencing-based transcriptomic, proteomic, metabolomic and electron microscopy approach to characterize multiple functional and metabolic changes induced by the Fur-mediated low iron response. Multiple cellular processes aside of iron transport including mainly energy metabolism, but also flagella formation and motility, cell wall architecture and antibiotic/CAMP resistance were controlled by iron and partly by Fur in *C. difficile*.

## Materials and Methods

### Bacterial Strains and Growth Conditions

*Escherichia coli* DH5a [*fhuA2 lac(del)U169 phoA glnV44 Φ80’ lacZ(del)M15 gyrA96 recA1 relA1 endA1 thi-1 hsdR17*], DH10B [F^-^ *mcrA* Δ(*mrr-hsd*RMS-*mcr*BC) Φ80d*lac*ZΔM15 Δ*lac*X74 *end*A1 *rec*A1 *deo*R Δ(*ara*,*leu*)7697 *ara*D139 *gal*U *gal*K *nup*G *rps*L λ^-^], and CA434 (*E*. *coli* HB101 carrying the Incβ conjugative plasmid R702) were grown in LB medium supplemented with 100 mg/l ampicillin or 20 mg/l chloramphenicol as required. *C. difficile* 630Δ*erm* cells were grown in Brain-Heart-Infusion (BHI) medium (37 g/l) supplemented with 0.1% L-cysteine and 5 mg/ml yeast extract. During mutagenesis experiments *C. difficile* supplement (250 mg/l D-cycloserine and 8 mg/l cefoxitin) (Sigma Aldrich, Taufkirchen, Germany) and 2.5 mg/l erythromycin (Carl Roth, Karlsruhe, Germany) were added. Growth and Omics experiments were performed in *Clostridium difficile* minimal medium (CDMM) (Neumann-Schaal et al., 2015) under anaerobic conditions using an anaerobic chamber from Coy Laboratories (Grass Lake, MI, United States). Different iron sources were tested as additives to the medium including 15 μM iron-sulfate, 15 μM iron-chloride, 15 μM iron-citrate, 10 μM hemin, 10 μg/ml ferritin and 10 μg/ml transferrin unless stated otherwise. Cells were harvested (10 min, 8,000 × *g*) anaerobically using gas-tight polypropylene tubes (TPP, Trasadingen, Switzerland) and harvested cells and/or supernatant were used for transcriptome, proteome and metabolome analyses as described below.

### Construction of the *C. difficile fur* Mutant and a *fur* Containing Vector for Complementation

The vector for *fur* inactivation was designed with the help of the ClosTron website^1^ using the Perutka algorithm (Perutka et al., 2004). *E. coli* CA434 was transformed with the resulting pMTL007C-E2_*fur*274a::intron vector (pMTL007C-E2 retargeted to CD630Δ*erm fur*274a::intron, *ermB*) for mating with *C. difficile* 630Δ*erm* cells after a standard protocol (Heap et al., 2007, 2009, 2010). The desired *fur* mutant was identified using primers Cdi-*fur*-F (5′-CTGGTTTTAAGATTACGCCAC-3′), Cdi-*fur*-R (5′-CCATTACACTCGTCACATAGTC-3′), EBSuni-versal (5′CGAAATTAGAAACTTGCGTTCAGTAAA-3′), Erm-RAM-RF (5′-ACGCGTTATATTGATAAAAATAATAGTGGG-3′), ErmRAM-R (5′-ACGCGTGCGACTCATAGAATTATTTCCTCCCG-3′) as described and documented in the Supplementary Material. For complementation of the *C. difficile fur* mutant, a PCR fragment covering the region from 300 bp upstream to 100 bp downstream of the *fur* gene (CD630_12870) was amplified using chromosomal *C. difficile* 630 DNA and the primers Cdi-*fur*-compl.NotI-F (5′-ATCAGCGGCCGCCAGATATTTATTATATTTGC-3′ and Cdi-*fur*-compl.HindIII-R (5′-ATCAAAGCTTAATGGAAGAATAGCATAG-3′) digested with NotI and HindIII and cloned into the appropriately cut shuttle vector pMTL82151 to generate pMTL82151_*fur* (Heap et al., 2009).

### Field Emission Scanning Electron Microscopy (FESEM)

*Clostridioides difficile* 630Δ*erm* and corresponding *fur* mutant were grown anaerobically in CDMM with and without addition of 15 μM iron-sulfate at 37°C to mid-exponential phase, harvested and fixated with 5% formaldehyde. Afterwards, the cells were washed with TE-buffer (20 mM TRIS, 1 mM EDTA, pH 6.9) before dehydration in a graded series of acetone (10, 30, 50, 70, and 90%) on ice for 15 min for each step. The 100% acetone dehydration step was performed at room temperature. Then, samples were critical-point dried with liquid CO_2_ (CPD 30, Bal-Tec, Balzers, Liechtenstein) and covered with a gold-palladium film by sputter coating (SCD 500, Bal-Tec, Balzers, Liechtenstein) before being examined in a field emission scanning electron microscope (Zeiss DSM 982 Gemini, Oberkochen, Germany) using the Everhart Thornley SE detector and the in lens detector in a 50:50 ratio at an acceleration voltage of 5 kV.

### RNA Sequencing

*Clostridioides difficile* 630Δ*erm* and corresponding *fur* mutant were grown anaerobically in CDMM with and without addition of 15 μM iron-sulfate at 37°C to mid-exponential phase and harvested. Employed CDMM without additions contained 0.2 μM iron. Due to the different growth behavior of both strains the mid-exponential growth rate was reached by both strains at different time points. At these two time points both strains revealed comparable growth rates. Total bacterial RNA was isolated from bacterial cell pellets as described before (Rosinski-Chupin et al., 2014). Residual DNA was removed using TURBO DNase (Ambion, Thermo Fisher Scientific, Waltham, MA, United States). Resulting DNA-free RNA was further purified with phenol:chloroform:isoamylalcohol (25:24:1) extraction. Remaining traces of phenol were removed by washing the samples twice with chloroform:isoamylalcohol (24:1). RNA integrity was assessed using the Agilent RNA 6000 Nano Kit on the Agilent 2100 Bioanalyzer (Agilent Technologies, Santa Clara, CA, United States). Transfer RNA was depleted from the total RNA using Microbexpress (Ambion, Thermo Fisher Scientific, Waltham, MA, United States). To 1 μg of rRNA depleted total RNA 1 μl of either 1:10 diluted ERCC ExFold RNA Spike-In Mix 1 or 2 (Ambion, Thermo Fisher Scientific, Waltham, MA, United States) was added. RNA was subsequently treated with tobacco acid pyrophosphatase (TAP) (Epicentre Biotechnologies, Madison, WI, United States). Prior to cDNA library preparation, RNA was further purified with phenol:chloroform:isoamylalcohol (25:24:1), any remaining phenol traces were removed by washing the samples twice with chloroform:isoamylalcohol (24:1), RNA was recovered by ethanol precipitation. Strand-specific RNA-Seq cDNA library preparation and barcode introduction based on RNA adapter ligation was performed as described previously (Nuss et al., 2015). Library quality was validated using the Agilent 2100 Bioanalyzer (Agilent Technologies, Santa Clara, CA, United States) following the manufacturer’s instruction. Cluster generation was performed using the Illumina cluster station. Single-end sequencing on the Illumina HiSeq2500 followed a standard protocol. The fluorescent images were processed to sequences and transformed to FastQ format using the Genome Analyzer Pipeline Analysis software 1.8.2 (Illumina, San Diego, CA, United States). The sequence output was controlled for general quality features. Sequencing adapter clipping and demultiplexing was done using the fastq-mcf and fastq-multxtool of ea-utils^2^. DNA sequencing output was analyzed using the FastQC tool (Babraham Bioinformatics, Cambridge, United Kingdom). All sequenced libraries were mapped to the *C. difficile* 630 genome (NC_009089.1) and the corresponding pCD630 plasmid (NC_008226.1) using Bowtie2 (version 2.1.0) (Langmead and Salzberg, 2012) with default parameters. ERCC mapping and analysis were performed after supplier’s instructions. After read mapping, SAMtools (Li et al., 2009) was employed to filter the resulting bam files for uniquely mapped reads (both strands), which were the basis for downstream analyses. Differential gene expression was evaluated using the DESeq2 tool as part of the Bioconductor software package. Throughout the manuscript the data were adapted to the *C. difficile* 630Δ*erm* annotation. In Table 1 both annotations are given. For the interconversion of the original data shown in Supplementary Tables S1–S3 and also the proteomics data in Supplementary Table S4 in the Supplemental Material an appropriate conversion table (Supplementary Table S5) is provided. The data discussed in this publication have been deposited in NCBI’s Gene Expression Omnibus (Edgar et al., 2002) and are accessible through GEO Series accession number GSE120189.

**Table 1:** Integration of the transcriptome (RNA-Seq), proteome, metabolome and bioinformatics-based Fnr –binding site analyses for the analysis of *C. difficile* to low iron conditions.

	Gene		Wild type: low vs.		*fur* mutant vs. wild type		Fur box
					
Locus_tag	name		high iron (log2 FC)		at high iron (log2 FC)		
			T	P	T	P	
**Metal transport systems**		
CD630_16470 CDIF630erm_01824	*yclN*	Iron family ABC transporter permease	4.79	0.18	5.90	–	+
CD630_16480 CDIF630erm_01825	*yclO*	Iron family ABC transporter permease	3.43	ON	4.74	ON	
CD630_16490 CDIF630erm_01826	*yclP*	Iron family ABC transporter ATP-binding protein	5.16	0.62	5.19	ON	
CD630_16500 CDIF630erm_01827	*yclQ*	Iron family ABC transporter substrate-binding protein	4.98	7.46	4.65	8.55	+
CD630_28740 CDIF630erm_03142		MATE family drug/sodium antiporter	2.42	–	4.12	–	
CD630_28750 CDIF630erm_03143	*fhuC*	Ferrichrome-specific ABC transporter ATP-binding protein	1.49	–	1.86	ON	
CD630_28760 CDIF630erm_03144	*fhuG*	Ferrichrome-specific ABC transporter permease	1.00	ON	3.85	ON	
CD630_28770 CDIF630erm_03145	*fhuB*	Ferrichrome-specific ABC transporter permease	1.15	ON	3.27	ON	+
CD630_28780 CDIF630erm_03146	*fhuD*	Ferrichrome-specific ABC transporter, substrate-binding	0.78	2.43	2.73	4.47	+
CD630_29890 CDIF630erm_03273	*ssuA2*	Sulfonate family ABC transporter substrate-binding protein	2.60	2.29	4.39	3.73	
CD630_29900 CDIF630erm_03274	*ssuB2*	Sulfonate family ABC transporter ATP-binding protein	2.91	3.09	4.50	4.81	
CD630_29910 CDIF630erm_03275	*ssuC2*	Sulfonate family ABC transporter permease	3.53	–	6.10	–	
CD630_29920 CDIF630erm_03276		Uncharacterized protein, iron hydrogenase-like	2.93	1.07	4.38	5.83	
CD630_10870 CDIF630erm_01231	*zupT*	Zinc transporter ZupT	1.85	ON	6.19	ON	+
CD630_05910 CDIF630erm_00704		ATPase	2.41	6.77	2.80	7.54	
CD630_05920 CDIF630erm_00705		Hypothetical protein	2.04	5.21	1.93	5.64	+
CD630_29970 CDIF630erm_03281		Iron family ABC transporter ATP-binding protein	1.02	0.19	1.08	1.16	
CD630_29980 CDIF630erm_03282		Iron family ABC transporter permease	0.42	0.06	1.95	–	
CD630_29990 CDIF630erm_03283		Iron family ABC transporter substrate-binding protein	0.17	-0.83	0.65	0.48	
CD630_14770 CDIF630erm_01641	*feoA*	Ferrous iron transport protein FeoA	1.51	ON	-1.18	ON	+
CD630_14780 CDIF630erm_01642	*feoA*	Ferrous iron transport protein FeoA1	1.02	5.70	-1.13	3.69	
CD630_14790 CDIF630erm_01643	*feoB1*	Ferrous iron transport protein FeoB1	0.77	7.19	-0.16	4.78	+
CD630_14800 CDIF630erm_01644		Hypothetical protein	0.40	–	-0.01	–	
CD630_15170 CDIF630erm_01684	*feoB*	Ferrous iron transport protein FeoB	-0.63	–	-0.92	–	
CD630_15180 CDIF630erm_01685	*feoA*	Ferrous iron transport protein FeoA	–	-	-0.14	–	
CD630_17451 CDIF630erm_01939	*feoA*	Ferrous iron transport protein	-2.34	0.36	-2.08	0.98	+
CD630_21680 CDIF630erm_02400	*hcp*	Hydroxylamine reductase	-0.81	0.29	-2.21	OFF	
CD630_21690 CDIF630erm_02401		Iron-sulfur binding protein	-1.86	–	-3.06	–	
CD630_03240 CDIF630erm_00452	*cbiM*	Cobalamin transport protein CbiM	-2.19	ON	-2.55	–	
CD630_03250 CDIF630erm_00453	*cbiN*	Cobalt ABC transporter substrate-binding protein CbiN	-1.81	–	-2.81	–	
CD630_03260 CDIF630erm_00454	*cbiQ*	Cobalt ABC transporter permease CbiQ	-2.05	ON	-1.13	ON	
CD630_03270 CDIF630erm_00455	*cbiO*	Cobalt ABC transporter ATP-binding protein CbiO	-0.47	-0.18	-1.81	-0.10	
**Pili and flagella formation**							
CD630_35040 CDIF630erm_03817		Type IV prepilin peptidase	-0.48	–	-0.33	–	
CD630_35050 CDIF630erm_03818		Twitching motility protein PilT	-0.91	-0.52	-1.71	0.12	
CD630_35060 CDIF630erm_03819		Hypothetical protein	-0.64	–	-2.01	–	
CD630_35070 CDIF630erm_03820		Type IV pilin	-0.29	–	-2.62	–	
CD630_35080 CDIF630erm_03821		Type IV pilin	-1.40	–	-1.74	–	
CD630_35090 CDIF630erm_03822		Type IV pilus assembly protein	-1.01	–	-2.43	–	
CD630_35100 CDIF630erm_03823		Membrane protein	-1.30	0.08	-1.91	-0.21	
CD630_35110 CDIF630erm_03824		Type IV pilus secretion protein	0.79	-1.43	-2.74	OFF	
CD630_35120 CDIF630erm_03825		Type IV pilus transporter system	-0.93	0.40	-2.50	-0.02	
CD630_35130 CDIF630erm_03826		Pilin protein	-1.61	0.21	-3.13	-0.52	03828
CD630_02520 CDIF630erm_00375	*fliJ*	Flagellar protein FliJ	2.37	ON	2.25	ON	
CD630_02530 CDIF630erm_00376	*fliK*	Flagellar hook-length control protein FliK	2.60	1.15	2.96	1.36	
CD630_02540 CDIF630erm_00377	*flgD*	Basal-body rod modification protein FlgD	2.68	–	2.41	–	
CD630_02550 CDIF630erm_00378	*flgE*	Flagellar hook protein FlgE	1.42	0.43	2.27	-0.18	
CD630_02551 CDIF630erm_00379	*FlbD*	Flagellar protein FlbD	3.26	1.56	1.28	0.91	
CD630_02560 CDIF630erm_00380	*motA*	Flagellar motor rotation protein MotA	2.38	-0.73	2.19	-0.39	
CD630_02570 CDIF630erm_00381	*motB*	Flagellar motor rotation protein MotB	1.97	-0.16	2.04	-0.79	
CD630_02580 CDIF630erm_00382	*fliL*	Flagellar basal body-associated protein FliL	1.84	0.53	2.60	0.24	
CD630_02590 CDIF630erm_00383	*fliZ*	Flagellar protein FliZ	1.05	ON	2.28	ON	
CD630_02600 CDIF630erm_00384	*fliP*	Flagellar biosynthesis protein FliP	1.80	OFF	3.06	-0.14	
CD630_02610 CDIF630erm_00385	*fliQ*	Flagellar biosynthetic protein FliQ	1.07	–	2.50	–	
CD630_02620 CDIF630erm_00386	*flhB*	Bifunctional flagellar biosynthesis protein FliR/FlhB	2.38	–	3.59	–	
CD630_02630 CDIF630erm_00387	*flhA*	Flagellar biosynthesis protein FlhA	3.84	0.18	4.44	0.10	
CD630_02640 CDIF630erm_00388	*flhF*	Flagellar biosynthesis regulator FlhF	3.61	1.04	3.84	1.30	
CD630_02650 CDIF630erm_00389	*flhG*	Flagellar biosynthesis protein FlhG	1.99	0.28	3.57	0.21	
CD630_02660 CDIF630erm_00390	*fliA*	Flagellar operon RNA polymerase sigma-28 factor	3.54	-0.01	4.15	OFF	
CD630_02670 CDIF630erm_00391		Flagellar protein	2.80	0.65	3.76	OFF	
CD630_02671		Flagellar protein	3.57	–	4.06	ON	
CD630_02680 CDIF630erm_00392	*flgG1*	Flagellar basal body rod protein FlgG	3.44	0.74	2.92	2.00	
CD630_02690 CDIF630erm_00393	*flgG*	Flagellar basal body rod protein FlgG	3.25	0.78	3.47	OFF	
CD630_02700 CDIF630erm_00394	*fliM*	Flagellar motor switch protein FliM	2.98	0.18	3.41	-0.12	
CD630_02710 CDIF630erm_00395	*fliN*	Flagellar motor switch phosphatase FliN	3.33	0.43	2.97	0.04	
CD630_02260 CDIF630erm_00348		Lytic transglycosylase	-3.23	0.78	-3.18	1.96	+
CD630_02270 CDIF630erm_00349		Hypothetical protein	-1.99	0.13	-3.99	-0.01	
CD630_02280 CDIF630erm_00350	*fliN*	Flagellar motor switch protein FliN	-2.25	0.25	-2.84	0.16	
CD630_02290 CDIF630erm_00351	*flgM*	Negative regulator of flagellin synthesis	-2.08	1.19	-3.40	OFF	
CD630_02300 CDIF630erm_00352		Flagellar biosynthesis protein	-2.41	0.92	-3.12	0.59	
CD630_02310 CDIF630erm_00353	*flgK*	Flagellar hook-associated protein FlgK	-2.08	1.58	-3.51	0.08	
CD630_02320 CDIF630erm_00354	*flgL*	Flagellar hook-associated protein FlgL	-2.21	0.22	-3.88	-0.46	
CD630_02330 CDIF630erm_00355	*fliW*	Flagellar assembly factor FliW	-2.66	-0.81	-3.16	0.15	
CD630_02340 CDIF630erm_00356	*crsA*	Carbon storage regulator CsrA	-1.87	0.09	-2.57	-0.46	
CD630_02350 CDIF630erm_00357	*fliS1*	Flagellar protein FliS1	-1.33	1.91	-3.04	1.89	
CD630_02360 CDIF630erm_00358	*fliS2*	Flagellar protein FliS2	-1.03	0.15	-2.30	0.20	
CD630_02370 CDIF630erm_00359	*fliD*	Flagellar hook-associated protein FliD	-0.94	1.65	-2.45	0.30	
CD630_02380 CDIF630erm_00360		Hypothetical protein	-0.69	1.30	-3.06	-0.56	
CD630_02390 CDIF630erm_00361	*fliC*	Flagellin C	-0.09	0.14	-1.57	-0.93	
CD630_22140 CDIF630erm_02447	*sinR*	HTH-type transcriptional regulator	1.85	OFF	2.46	0.72	
CD630_22150 CDIF630erm_02448		HTH-type transcriptional regulator	2.17	–	2.68	–	02449
CD630_19970 CDIF630erm_02215		Proline iminopeptidae	2.04	0.26	4.07	1.63	
CD630_19980 CDIF630erm_02216		TetR family transcriptional regulator	1.85	ON	3.73	ON	

**Polyamine biosynthesis and transport**							

CD630_08880 CDIF630erm_01008	*speA*	Arginine decarboxylase	3.92	ON	4.48	ON	+
CD630_08890 CDIF630erm_01009	*speH*	*S*-adenosylmethionine decarboxylase	2.57	ON	2.95	ON	
CD630_08900 CDIF630erm_01010	*speE*	Polyamine aminopropyl transferase	4.53	0.38	3.78	0.59	
CD630_08910 CDIF630erm_01011	*speB*	Agmatinase	5.41	-0.31	4.95	0.53	
CD630_10230 CDIF630erm_01159		Transcriptional regulator	3.64	-0.32	2.21	0.66	
CD630_10240 CDIF630erm_01160	*potA*	Spermidine/putrescine ABC transporter ATP-binding protein	3.43	-0.06	3.05	0.43	
CD630_10250 CDIF630erm_01161	*potB*	Spermidine/putrescine ABC transporter permease	3.24	-0.66	3.22	-0.66	
CD630_10260 CDIF630erm_01162	*potC*	Spermidine/putrescine ABC transporter permease	2.98	–	3.23	–	
CD630_10270 CDIF630erm_01163	*potD*	Spermidine/putrescine ABC transporter substrate-binding protein	2.57	-0.58	2.94	1.78	

**Antibiotic/CAMP resistance and cell wall restructuring**							

CD630_28510 CDIF630erm_03118	*dltC*	D-alanine–poly(phosphoribitol) ligase subunit 2	1.51	–	2.10	–	
CD630_28520 CDIF630erm_03119	*dltB*	D-alanyl transferase DltB	2.84	OFF	2.79	OFF	
CD630_28530 CDIF630erm_03120	*dltA*	D-alanine–poly(phosphoribitol) ligase subunit 1	1.37	-0.80	1.68	-1.19	
CD630_28540 CDIF630erm_03122	*dltD*	D-alanine transferase DltD	1.49	0.13	1.37	0.01	
CD630_16260 CDIF630erm_01803	*vanG*	D-alanyl-alanine synthetase A	-1.00	–	2.91	–	
CD630_16270 CDIF630erm_01804	*vanY*	D-alanyl-D-alanine carboxypeptidase	-0.89	–	3.13	–	
CD630_16280 CDIF630erm_01805	*vanTG*	Alanine racemase 1	0.64	–	3.47	–	
CD630_03160 CDIF630erm_00443		ABC transporter permease	1.10	–	2.42	–	
CD630_03170 CDIF630erm_00444		ABC transporter permease	0.67	–	2.61	–	
CD630_03180 CDIF630erm_00445		Bacitracin/multidrug family ABC transporter ATP-binding protein	1.51	-0.19	2.85	0.45	
CD630_08200 CDIF630erm_00938		Two-component response regulator	0.72	ON	1.58	ON	
CD630_08210 CDIF630erm_00939		Two-component sensor histidine kinase	1.39	ON	2.76	ON	
CD630_08220 CDIF630erm_00940		Multidrug family ABC transporter ATP-binding protein	1.04	–	2.35	–	
CD630_08230 CDIF630erm_00941		Multidrug family ABC transporter permease	-0.38	–	1.62	–	
CD630_08240 CDIF630erm_00943		Multidrug family ABC transporter permease	0.78	–	3.41	–	
CD630_20030 CDIF630erm_02221	*effD*	MATE family drug/sodium antiporter	-3.56	–	-1.20	–	
CD630_20040 CDIF630erm_02222	*effR*	MarR family transcriptional regulator	-5.25	-0.75	-2.98	3.12	
CD630_15570 CDIF630erm_01726		Peptidyl-prolyl isomerase	1.07	-0.58	3.69	0.16	+
CD630_15580 CDIF630erm_01727	*csfV*	ECF RNA polymerase sigma factor CsfV	2.29		3.42	–	
CD630_15590 CDIF630erm_01728	*rsiV*	Anti ECF RNA polymerase sigma factor RsiV	1.55	–	2.73	–	
CD630_26510 CDIF630erm_02905	*murG*	UDP-*N*-acetylglucosamine-*N*-acetylmuramyl-(pentapeptide) pyrophosphoryl-undecaprenol *N*- acetylglucosamine transferase	1.30	0.63	1.54	1.34	
CD630_26520 CDIF630erm_02906	*spoVE*	Cell division/stage V sporulation protein	0.91	–	2.04	–	
CD630_26530 CDIF630erm_02907	*murD*	UDP-*N*-acetylmuramoylalanine–D-glutamate ligase	0.77	0.11	1.45	0.42	
CD630_26540 CDIF630erm_02908	*mraY*	Phospho-*N*-acetylmuramoyl-pentapeptide-transferase	0.82	–	2.37	–	
CD630_26550 CDIF630erm_02909	*murF*	UDP-*N*-acetylmuramoyl-tripeptide–D-alanyl-D-alanine ligase	2.59	-0.26	2.74	0.21	
CD630_27780 CDIF630erm_03041		Glycosyl transferase family protein	0.98	-0.10	1.26	0.10	
CD630_27790 CDIF630erm_03042	*manC*	Mannose-1-phosphate guanylyltransferase	1.33	0.81	2.47	0.33	
CD630_27800 CDIF630erm_03043	*pgm*	Phosphoglucomutase	2.75	0.22	3.20	0.06	
CD630_27810 CDIF630erm_03044	*mviN*	Transmembrane virulence factor	3.67	-0.93	4.13	0.78	
CD630_02840 CDIF630erm_00408		PTS system mannose/fructose/sorbose transporter subunit IIA	–	-0.04	–	0.88	
CD630_02850 CDIF630erm_00409		PTS system mannose/fructose/sorbose transporter subunit IIB	1.86	0.14	2.61	0.62	
CD630_02860 CDIF630erm_00410		PTS system mannose/fructose/sorbose transporter subunit IIA	2.27	-0.52	2.43	0.64	
CD630_02870 CDIF630erm_00411		PTS system mannose/fructose/sorbose transporter subunit IIB	1.90	0.20	2.70	1.00	
CD630_02880 CDIF630erm_00412		PTS system mannose/fructose/sorbose transporter subunit IIC	1.51	-0.14	2.68	0.06	
CD630_02890 CDIF630erm_00413		PTS system mannose/fructose/sorbose transporter subunit IID	1.90	-0.08	1.68	0.46	
CD630_32070 CDIF630erm_03501		Multi antimicrobial extrusion protein	1.30	–	2.93	–	
CD630_32080 CDIF630erm_03502		MarR family transcriptional regulator	0.99	ON	2.77	ON	
CD630_35140 CDIF630erm_03828	*prs*	Ribose-phosphate pyrophosphokinase	1.89	-0.10	2.31	0.30	
CD630_35150 CDIF630erm_03829	*glmU*	Bifunctional *N*-acetylglucosamine-1- phosphate uridyltransferase/glucosamine-1-phosphate acetyltransferase	1.51	-0.27	2.04	0.40	+
CD630_10090 CDIF630erm_01145		GntR family transcriptional regulator	-3.62	–	-2.36	–	
CD630_10100 CDIF630erm_01146	*nagA*	*N*-acetylglucosamine-6-phosphate deacetylase	-2.69	-2.60	-2.02	-0.22	
CD630_10110 CDIF630erm_01147	*nagB*	Glucosamine-6-phosphate deaminase	-2.74	1.69	-1.74	0.19	

**Energy metabolism**							

CD630_32370 CDIF630erm_03533	*prdF*	Proline racemase	3.03	-1.48	4.04	-1.14	
CD630_32380 CDIF630erm_03534		Proline reductase PrdE-like protein	4.31	–	4.34	–	
CD630_32390 CDIF630erm_03535	*prdE*	Proline reductase PrdE	4.14	-2.05	4.48	-2.14	
CD630_32400 CDIF630erm_03536	*prdD*	Proline reductase PrdD	4.18	-1.83	4.88	-1.00	
CD630_32410 CDIF630erm_03537	*prdB*	Proline reductase	1.74	-0.53	2.69	-0.80	
CD630_32430 CDIF630erm_03539		Hypothetical protein	2.90	-0.79	2.07	-0.39	
CD630_32440 CDIF630erm_03540	*prdA*	D-proline reductase PrdA	2.98	-0.67	3.05	-0.83	
CD630_32450 CDIF630erm_03541	*prdR*	Sigma-54 dependent transcriptional regulator	0.61	0.02	-1.02	-0.80	
CD630_32460 CDIF630erm_03542		Surface protein	-1.35	–	-2.85	–	
CD630_32470 CDIF630erm_03544	*prdC*	Electron transfer protein	4.62	-0.96	4.78	-0.90	
CD630_11370 CDIF630erm_01284	*rnfC*	Electron transport complex protein RnfC	2.64	-0.73	2.91	-0.49	
CD630_11380 CDIF630erm_01285	*rnfD*	Electron transport complex protein RnfD	2.01	-1.19	3.58	-0.62	
CD630_11390 CDIF630erm_01286	*rnfG*	Electron transport complex protein RnfG	2.67	0.47	3.31	0.41	
CD630_11400 CDIF630erm_01287	*rnfE*	Electron transport complex protein RnfE	1.17	OFF	3.47	OFF	
CD630_11410 CDIF630erm_01288	*rnfA*	Electron transport complex protein RnfA	1.88	–	3.55	–	
CD630_11420 CDIF630erm_01289	*rnfB*	Electron transport complex protein RnfB	1.61	-1.38	2.47	-0.91	
CD630_11700 CDIF630erm_01318	*larA*	Lactate racemase	1.41	–	2.86	–	
CD630_11710 CDIF630erm_01319	*etfB*	Lactate dehydrogenase, electron transfer flavoprotein beta subunit	0.67	OFF	2.43	OFF	
CD630_11720 CDIF630erm_01320	*etfA*	Lactate dehydrogenase, electron transfer flavoprotein alpha subunit	-0.66	–	0.63	–	
CD630_11730 CDIF630erm_01321		lactate dehydrogenase (electron bifurcating), catalytic subunit	-0.30	-0.57	0.73	OFF	
CD630_03940 CDIF630erm_00522	*ldhA*	(R)-2-hydroxyisocaproate dehydrogenase	-8.37	OFF	-8.66	OFF	
CD630_03950 CDIF630erm_00523	*hadA*	Isocaprenoyl-CoA:2-hydroxyisocaproate CoA-transferase	-8.33	OFF	-11.10	OFF	00519
CD630_03960 CDIF630erm_00524	*hadI*	2-hydroxyisocaproyl-CoA dehydratase activator	-0.79	OFF	-11	OFF	
CD630_03970 CDIF630erm_00525	*hadB*	Oxygen-sensitive 2-hydroxyisocaproyl-CoA dehydratase subunit B	-7.47	OFF	-10.52	OFF	
CD630_03980 CDIF630erm_00526	*hadC*	Oxygen-sensitive 2-hydroxyisocaproyl-CoA dehydratase subunit C	-7.81	-4.86	-10.94	OFF	
CD630_03990 CDIF630erm_00527	*acdB*	Acyl-CoA dehydrogenase	-7.43	-4.27	-10.17	-4.76	
CD630_04000 CDIF630erm_00528	*etfB1*	Electron transfer flavoprotein subunit beta	-7.28	-3.90	-10.48	-4.50	
CD630_04010 CDIF630erm_00529	*etfA1*	Electron transfer flavoprotein subunit alpha	-7.50	-3.90	-10.39	-4.98	
CD630_10540 CDIF630erm_01194	*bcd2*	Butyryl-CoA dehydrogenase	-5.89	OFF	-8.00	OFF	
CD630_10550 CDIF630erm_01195	*etfB*	Electron transfer flavoprotein subunit beta	-7.47	OFF	-7.70	OFF	
CD630_10560 CDIF630erm_01196	*etfA*	Electron transfer flavoprotein subunit alpha	-6.75	OFF	-7.75	OFF	
CD630_10570 CDIF630erm_01197	*crt2*	3-hydroxybutyryl-CoA dehydratase	-6.37	–	-7.48	OFF	
CD630_10580 CDIF630erm_01198	*hbd*	3-hydroxybutyryl-CoA dehydrogenase	-5.21	OFF	-5.97	OFF	
CD630_10590 CDIF630erm_01199	*thlA1*	Acetyl-CoA acetyltransferase	-6.22	OFF	-6.43	OFF	
CD630_29660 CDIF630erm_03250	*adhE*	Bifunctional acetaldehyde-CoA/alcohol dehydrogenase	-8.19	-4.52	-9.86	-5.91	
CD630_23380 CDIF630erm_02577	*4hbD*	4-hydroxybutyrate dehydrogenase	-1.00	-0.87	-1.23	-1.06	02575
CD630_23390 CDIF630erm_02578	*cat2*	4-hydroxybutyrate CoA-transferase	-0.87	OFF	-0.50	OFF	
CD630_23400 CDIF630erm_02579		Hypothetical protein	-2.16	–	-1.55	–	
CD630_23410 CDIF630erm_02580	*abfD*	Gamma-aminobutyrate metabolism dehydratase/isomerase	-1.41	-1.74	-1.23	-1.16	
CD630_23420 CDIF630erm_02581	*sucD*	Succinate-semialdehyde dehydrogenase	-2.47	–	-3.77	–	
CD630_23430 CDIF630erm_02582	*cat1*	Succinyl-CoA:coenzyme A transferase	-3.35	–	-3.58	–	
CD630_23440 CDIF630erm_02583		Membrane protein	-4.79	–	-5.28	–	
CD630_23480 CDIF630erm_02587	*grdD*	Glycine reductase complex component D	–	OFF	–	OFF	
CD630_23490 CDIF630erm_02588	*grdC*	Glycine reductase complex component C	-3.70	–	-3.70	–	
CD630_23510 CDIF630erm_02589	*grdB*	Glycine reductase complex component B	–	OFF	–	OFF	
CD630_23520 CDIF630erm_02592	*grdA*	Glycine reductase complex component A	–	-	–	-	
CD630_23540 CDIF630erm_02594	*grdE*	Glycine reductase complex component E	-5.02	-2.84	-5.22	OFF	
CD630_23550 CDIF630erm_02595	*trxA2*	Thioredoxin 2	-3.66	–	-3.66	–	+
CD630_23560 CDIF630erm_02596	*trxB3*	Thioredoxin reductase	–	-	–	OFF	
CD630_23570 CDIF630erm_02597	*grdDX*		–	OFF	–		
CD630_08530 CDIF630erm_00972	*oppB*	Oligopeptide family ABC transporter permease	-2.81	OFF	-2.26	OFF	
CD630_08540 CDIF630erm_00973	*oppC*	Oligopeptide family ABC transporter permease	-2.67	–	-2.28	–	
CD630_08550 CDIF630erm_00974	*oppA*	Oligopeptide family ABC transporter substrate-binding protein	-2.48	-0.37	-3.22	-1.02	
CD630_08560 CDIF630erm_00975	*oppD*	ABC transporter ATP-binding protein	-2.38	–	-3.65	–	
CD630_15360 CDIF630erm_01704	*nfnA*	NADH-dependent reduced ferredoxin:NADP oxidoreductase	-2.56	-1.18	-2.43	-0.71	
CD630_15370 CDIF630erm_01705	*nfnB*	NADH-dependent reduced ferredoxin:NADP oxidoreductase	-2.69	-1.77	-3.01	-1.53	
CD630_08820 CDIF630erm_01002	*glgC*	Glucose-1-phosphate adenylyltransferase	-1.89	–	-3.38	–	
CD630_08830 CDIF630erm_01003	*glgD*	Glycogen biosynthesis protein	-1.70	-2.42	-4.22	OFF	
CD630_08840 CDIF630erm_01004	*glgA*	Glycogen synthase	-2.45	1.98	-3.24	-0.37	
CD630_08850 CDIF630erm_01005	*glgP*	Glycogen phosphorylase	-1.62	0.49	-3.38	0.14	
CD630_08860 CDIF630erm_01006	*amyB*	Amylopullulanase	-0.12	-0.29	-2.68	0.05	
CD630_23180 CDIF630erm_02556		Phosphohexomutase	2.01	0.58	1.56	0.21	
CD630_23190 CDIF630erm_02557	*rpe*	Ribulose-phosphate 3-epimerase	1.24	ON	1.19	ON	
CD630_23200 CDIF630erm_02558	*rpiB1*	Ribose-5-phosphate isomerase B	3.19	0.00	2.23	-0.02	
CD630_23210 CDIF630erm_02559		Transketolase	1.75	0.44	2.01	0.22	
CD630_23220 CDIF630erm_02560	*tkt*	Transketolase	1.48	0.23	2.63	0.29	
CD630_11200 CDIF630erm_01264	*pflD*	Formate acetyltransferase	-0.51	-0.12	2.03	1.70	
CD630_11210 CDIF630erm_01265	*pflC*	Pyruvate formate-lyase activating enzyme	-0.32	–	2.21	–	
CD630_11220 CDIF630erm_01266		Transcriptional regulator	-1.51	-0.63	1.48	0.05	
CD630_32820 CDIF630erm_03582	*pflD*	Pyruvate formate-lyase	-1.38	-2.38	-2.88	-2.94	
CD630_32830 CDIF630erm_03583	*pflE*	Pyruvate formate-lyase	-2.11	–	-3.53	–	
CD630_33130 CDIF630erm_03614	*hydN1*	Oxidoreductase Fe-S subunit	-2.44	–	-3.60	–	
CD630_33140 CDIF630erm_03615	*hydA*	Iron hydrogenase	-2.59	OFF	-3.08	-1.30	
CD630_33150 CDIF630erm_03616	*hydN2*	Oxidoreductase Fe-S subunit	-3.54	-1.13	-3.00	-0.86	+
CD630_33151 CDIF630erm_03617		Hypothetical protein	-1.53	–	-3.00	–	
CD630_33160 CDIF630erm_03618	*fdhD*	Formate dehydrogenase accessory protein FdhD	-2.22	–	-3.03	–	
CD630_33170 CDIF630erm_03619	*fdhF*	Formate dehydrogenase-H	-2.88	-1.63	-3.37	-1.72	
CD630_01740 CDIF630erm_00296	*cooS*	Carbon monoxide dehydrogenase	-3.57	-1.46	-4.70	-2.05	
CD630_01750 CDIF630erm_00297	*cooF*	Oxidoreductase Fe-S subunit	-2.43	–	-4.33	–	
CD630_01760 CDIF630erm_00298		Oxidoreductase NAD/FAD binding subunit	-2.12	OFF	-3.96	OFF	
CD630_12780 CDIF630erm_01431	*iscR*	Rrf2 family transcriptional regulator	4.43	3.30	-0.27	1.44	
CD630_12790 CDIF630erm_01432	*iscS2*	Cysteine desulfurase	0.55	2.69	-3.25	0.03	
CD630_12800 CDIF630erm_01433		NifU family iron-sulfur cluster assembly protein	0.83	3.83	-2.97	0.31	
CD630_34670 CDIF630erm_03778	*atpC*	ATP synthase subunit epsilon	3.83	0.56	1.37	1.04	
CD630_34680 CDIF630erm_03779	*atpD*	ATP synthase subunit beta	2.25	0.60	2.04	0.94	
CD630_34690 CDIF630erm_03780	*atpG*	ATP synthase subunit gamma	3.91	0.51	2.83	0.55	
CD630_34700 CDIF630erm_03781	*atpA*	ATPase subunit alpha	3.72	0.61	2.44	1.00	
CD630_34710 CDIF630erm_03782	*atpH*	ATP synthase subunit delta	3.65	0.49	2.67	0.72	
CD630_34720 CDIF630erm_03783	*atpF*	ATP synthase subunit B	3.33	0.16	2.31	0.47	
CD630_34730 CDIF630erm_03784	*atpE*	ATP synthase subunit C	2.81	ON	1.58	ON	
CD630_34740 CDIF630erm_03785	*atpB*	ATP synthase subunit A	1.59	–	1.77	0.94	
CD630_29540 CDIF630erm_03237	*atpD*	V-type ATP synthase subunit D	-1.47	–	-2.46	–	
CD630_29550 CDIF630erm_03238	*atpB*	V-type ATP synthase subunit B	-3.00	-0.98	-2.58	-1.34	
CD630_29560 CDIF630erm_03239	*atpA*	V-type ATP synthase subunit A	-2.35	-0.73	-2.54	-0.95	+
CD630_29561 CDIF630erm_03240	*atpF*	V-type ATP synthase subunit F	-2.40	–	-1.52	–	
CD630_29570 CDIF630erm_03241	*atpC*	V-type ATP synthase subunit C	-2.32	–	-2.56	–	
CD630_29580 CDIF630erm_03242	*atpE*	V-type ATP synthase subunit E	-1.47	OFF	-2.92	OFF	
CD630_29590 CDIF630erm_03243	*atpK*	V-type ATP synthase subunit K	-1.47	-2.10	-2.48	-1.20	
CD630_29600 CDIF630erm_03244	*atpI*	V-type ATP synthase subunit I	-2.73	OFF	-3.24	OFF	
CD630_29610 CDIF630erm_03245		Hypothetical protein	-1.78	0.00	-2.92	-0.80	

**Flavodoxin formation**							

CD630_19990 CDIF630erm_02217	*fldx*	Flavodoxin	4.54	ON	4.54	ON	
CD630_16970 CDIF630erm_01882	*ribH*	6.7-dimethyl-8-ribityllumazine synthase	2.82	0.31	3.86	0.68	+
CD630_16980 CDIF630erm_01883	*ribBA*	Riboflavin biosynthesis bifunctional 3.4-dihydroxy-2-butanone 4-phosphate synthase/GTP cyclohydrolase	2.10	0.40	2.62	0.99	
CD630_16990 CDIF630erm_01884	*ribE*	Riboflavin synthase subunit alpha	1.82	0.26	1.55	-0.07	
CD630_17000 CDIF630erm_01885	*ribD*	Riboflavin biosynthesis bifunctional diaminohydroxyphosphoribosylamino pyrimidine deaminase/5-amino-6-(5-phosphoribosylamino)uracil reductase	1.39	0.35	1.81	1.02	
CD630_23310 CDIF630erm_02570	*mtlD*	Mannitol-1-phosphate 5-dehydrogenase	-6.28	-2.60	-5.79	-1.91	
CD630_23320 CDIF630erm_02571	*mtlF*	PTS system mannitol-specific transporter subunit IIA	-5.98	OFF	-5.53	-1.54	
CD630_23330 CDIF630erm_02572	*mtlR*	PTS operon transcription antiterminator	-5.78	OFF	-6.17	OFF	
CD630_23340 CDIF630erm_02573	*mtlA*	PTS system mannitol-specific transporter subunit IICB	-6.29	OFF	-5.92	-3.14	

**Fatty acid metabolism**							

CD630_11770 CDIF630erm_01326	*fapR*	Fatty acid biosynthesis transcriptional regulator	2.53	0.17	3.16	0.11	
CD630_11780 CDIF630erm_01327	*plsX*	Phosphate acyltransferase	1.93	-1.29	3.25	-0.22	
CD630_11790 CDIF630erm_01328	*fabH*	3-oxoacyl-ACP synthase	2.12	0.09	4.00	0.92	
CD630_11800 CDIF630erm_01329	*fabK*	Enoyl-(acyl-carrier-protein) reductase II	2.86	-0.16	3.61	0.50	
CD630_11810 CDIF630erm_01330	*fabD*	Malonyl CoA-acyl carrier protein transacylase	3.03	-0.18	3.87	0.41	
CD630_11820 CDIF630erm_01331	*fabG*	3-oxoacyl-ACP reductase	3.65	-0.06	3.94	0.23	
CD630_11830 CDIF630erm_01332	*acpP*	Acyl carrier protein	2.95	–	4.28	–	
CD630_11840 CDIF630erm_01333	*fabF*	3-oxoacyl-ACP synthase	2.98	0.07	4.71	0.69	01334

**CRISPR/Cas and prophages**							

CD630_29750 CDIF630erm_03259		CRISPR-associated endoribonuclease Cas2	-0.74	–	-2.13	–	
CD630_29760 CDIF630erm_03260		CRISPR-associated endonuclease Cas1	-0.87	–	-1.67	–	
CD630_29770 CDIF630erm_03261		CRISPR-associated Cas4 family protein	-0.41	–	-2.72	–	
CD630_29780 CDIF630erm_03262		CRISPR-associated Cas3 family helicase	-0.26	–	-2.20	–	
CD630_29790 CDIF630erm_03263		CRISPR-associated Cas5 family protein	-1.30	–	-1.66	–	
CD630_29800 CDIF630erm_03264		CRISPR-associated autoregulatorDevR family protein	0.20	-0.55	-1.97	-1.28	
CD630_29810 CDIF630erm_03265		CRISPR-associated protein	-1.27	-1.20	-1.78	OFF	
CD630_29820 CDIF630erm_03266		CRISPR-associated Cas6 family protein	-0.60	–	-2.92	–	
CD630_13650 CDIF630erm_01522		XkdN-like protein	–	-	-2.30	–	+
CD630_13660 CDIF630erm_01524		Tail protein	-1.33	ON	-3.28	–	
CD630_13680 CDIF630erm_01526		Cell wall XkdQ-like hydrolase	0.02	–	-3.93	–	
CD630_13700 CDIF630erm_01528		XkdS-like protein	–	-	-1.07	–	
CD630_13710 CDIF630erm_01529		Baseplate assembly protein	-1.33	–	-2.06	–	
CD630_13720 CDIF630erm_01530		XkdT-like protein	-1.27	–	-2.90	–	
CD630_13740 CDIF630erm_01532		Beta-lactamase-inhibitor protein II	0.64	ON	-1.58	–	

**DNA/RNA nucleotide metabolism**							

CD630_02180 CDIF630erm_00340	*purE*	5-carboxyaminoimidazole ribonucleotide mutase	0.09	-0.24	2.12	0.80	
CD630_02190 CDIF630erm_00341	*purC*	Phosphoribosylaminoimidazolesuccino carboxamide synthase	-0.22	-0.21	2.59	0.41	
CD630_02200 CDIF630erm_00342	*purF*	Amidophosphoribosyltransferase	1.65	-0.69	2.96	0.09	
CD630_02210 CDIF630erm_00343	*purG*	Phosphoribosylformylglycinamidinecyclo-ligase	0.60	-0.76	2.94	-0.69	
CD630_02220 CDIF630erm_00344	*purN*	Phosphoribosylglycinamideformyl transferase	2.66	-0.41	3.49	-0.04	
CD630_02230 CDIF630erm_00345	*purH*	Bifunctional phosphoribosylaminoimidazole carboxamideformyltransferase/IMP cyclohydrolase	2.49	-0.08	3.37	0.24	
CD630_02240 CDIF630erm_00346	*purD*	Phosphoribosylamine–glycine ligase	2.19	-0.28	3.07	0.32	
CD630_02250 CDIF630erm_00347	*purL*	Phosphoribosylformylglycinamidine synthase	1.24	-0.06	2.23	0.19	00348
CD630_01840 CDIF630erm_00305	*pyrB*	Aspartate carbamoyltransferase	0.19	0.47	1.01	0.60	
CD630_01850 CDIF630erm_00306	*pyrK*	Dihydroorotate dehydrogenase electron transfer subunit	2.94	0.82	2.82	0.74	
CD630_01860 CDIF630erm_00307	*pyrD*	Dihydroorotate dehydrogenase 1B	1.96	0.21	2.72	0.16	
CD630_01870 CDIF630erm_00308	*pyrE*	Orotatephosphoribosyltransferase	3.92	0.19	3.38	0.54	
CD630_25940 CDIF630erm_02848	*uraA*	Uracil-specific ABC transporter permease	0.96	0.17	3.83	-0.46	
CD630_25950 CDIF630erm_02849	*pyrR*	Bifunctional pyrimidine operon regulatory protein/uracil phosphoribosyltransferase	–	0.05	2.17	0.62	+
CD630_25960 CDIF630erm_02850		Pseudouridylate synthase	1.85	–	2.05	–	
CD630_25970 CDIF630erm_02851	*lspA*	Lipoprotein signal peptidase	2.47	–	3.02	–	
CD630_27690 CDIF630erm_03032		Polysaccharide biosynthesis protein	1.95	0.29	2.73	-0.39	
CD630_27700 CDIF630erm_03033		Group 1 glycosyl transferase	1.22	-0.32	2.55	0.20	
CD630_27710 CDIF630erm_03034	*rkpK*	UDP-glucose 6-dehydrogenase	2.13	-0.05	2.59	0.41	
CD630_27720 CDIF630erm_03035	*tuaG*	Family 2 glycosyl transferase	2.23	-0.23	3.25	0.35	
CD630_27730 CDIF630erm_03036		Family 2 glycosyl transferase	2.45	-0.53	2.59	0.06	
CD630_27740 CDIF630erm_03037		Family 2 glycosyl transferase	1.24	-0.52	2.30	0.64	
CD630_27750 CDIF630erm_03038		Glycerophosphotransferase	2.64	-0.61	2.73	-0.07	
CD630_27760 CDIF630erm_03039		Family 2 glycosyl transferase	0.78	-0.06	1.99	0.17	


*T, transcriptome, P, proteome, FC, fold change, shown are corresponding locus tags of C. difficile 630 [according to (Monot et al., 2011)] and 630Δerm [according to (Dannheim et al., 2017a)]. The gene number in the Fur column identifies a gene close to the regulated genes shown in the table possessing a potential Fur box in its upstream region. ON and OFF describe proteomics result, where under one of the two compared conditions/strains no protein was detected. If the first protein is detectable under conditions of interest, it is ON, if it is absent, it is OFF.*

### Proteomics

Bacteria were grown as outlined for the RNA-seq experiments. Cell pellets were suspended in 700 μl of ice-cold urea-containing buffer (7 M urea, 2 M thiourea, 50 mM dithiothreitol (DTT), 4% (w/v) 3-[(3-cholamidopropyl) dimethylammonio]-1-propanesulfonate (CHAPS), 50 mM Tris-HCl). Cell lysis was performed by sonication (probe MS73, Sonoplus, Bandelin, Berlin, Germany) in six cycles of 60s (amplitude 60%, 0.1 s pulse every 0.5 s) on ice. Cell debris was removed by centrifugation at 6,000 *g* for 10 min at 4°C. Proteins of cell free resulting lysates were precipitated by addition of ice-cold acetone [in a 1:5 ratio (v/v)] for 20 h at -20°C. Subsequently, samples were allowed to warm to room temperature and were centrifuged at 22,000 *g* for 45 min at room temperature. The supernatant was discarded, the protein pellets were washed in 80% acetone, and subsequently in 100% acetone, before they were air-dried. The protein pellets were solubilized in SDS-containing urea-buffer [7 M urea, 2 M thiourea, 1% (v/v) SDS]. For protein concentration determination, 10 μl of each sample was separated by SDS-PAGE (Criterion TGX Precast Gels 4–20%, Bio-Rad, Hercules, CA, United States). Resulting SDS-gels were fixed for 1 h in 40% (v/v) EtOH, 10% (v/v) glacial acidic acid, washed in H_2_O and stained by the Flamingo fluorescent dye (Bio-Rad, Hercules, CA, United States) for 1 h. Resulting fluorescence signals of the samples were measured by a Typhoon TRIO scanner (GE Healthcare, Little Chalfont, United Kingdom), quantified by ImageQuant 5.2 (GE Healthcare, Little Chalfont, United Kingdom) and used for quantitative normalization of protein. Comparable protein amounts (∼30 μg of protein per sample) for each analyzed condition were separated by SDS-PAGE as described above and stained overnight with Colloidal Coomassie. Gel lanes were cut into 10 slices and proteins subjected to in-gel trypsinization as described previously (Lassek et al., 2015).

The eluted peptides were subjected to LC-MS/MS analyses performed on a Proxeon nLC 1000 coupled online to an Orbitrap Elite mass spectrometer (Thermo Fisher Scientific, Waltham, MA, United States). In-house self-packed columns [i.d. 100 μm, o.d. 360 μm, length 150 mm; packed with 1.7 μm Aeris XB-C18 reversed-phase material (Phenomenex, Aschaffenburg, Germany)] were loaded and washed with 10 μl of buffer A [0.1% (v/v) acetic acid] at a maximum pressure of 750 bar. For coupled LC-MS/MS analysis, elution of peptides took place with a non-linear 80 min gradient from 1 to 99% buffer B [0.1% (v/v) acetic acid in acetonitrile] at a constant flow rate of 300 nl/min. Eluting peptides were recorded in the mass spectrometer at a resolution of *R* = 60,000 with lockmass correction activated. After acquisition of the full MS spectra, up to 20 dependent scans (MS/MS) were performed according to precursor intensity by collision-induced dissociation fragmentation (CID) in the linear ion trap. For protein identification and quantification from raw MS data, the Proteome Discoverer^TM^ software (version 1.4, Thermo Fisher Scientific Inc., Waltham, MA, United States) was used, and results further evaluated employing Scaffold (version 4.4, Proteome Software Inc., Portland, OR, United States) as previously described in detail (Lassek et al., 2015). In brief, Sequest HT database searches were performed with raw files against a *C. difficile* 630 protein database containing common contaminations (3804 entries). The following search parameters were used: enzyme type = trypsin (KR), peptide tolerance = 10 ppm, tolerance for fragment ions = 0.6 Da, b- and y-ion series, variable modification = methionine (15.99 Da); a maximum of three modifications per peptide was allowed. Peptide and protein identifications were accepted with a false discovery rate (FDR) of at most 1%, requiring a minimum of at least two unique peptides for protein identification and quantification. Moreover, only proteins that were at least identified in two out of three biological replicates were taken into account. Relative protein quantification was achieved by calculating the normalized area under the curve (NAUC). Identification of statistical differences in relative protein amounts was performed using *t*-test (*p*-value < 0.05) including adjusted Bonferroni correction and all possible permutations. Proteome data are summarized in Supplementary Table S4. Data of interest can be easily converted into the *C. difficile* 630Δ*erm* annotation using conversion Supplementary Table S5. All MS raw data as well as Proteome Discoverer and Scaffold result files have been deposited to the ProteomeXchange Consortium via the PRIDE partner repository (Vizcaino et al., 2016) with data set identifier PXD011161.

### Metabolomics

Cells were grown to the mid-exponential growth phase and harvested anaerobically as indicated above for the transcriptome and proteome experiments. The supernatant was removed and the cells were immediately quenched by suspension in pre-cooled isotonic sodium chloride-methanol [50% (v/v), -32°C]. Cells were pelleted at -20°C, 8,000 *g* for 5 min. The quenching solution was removed and the cells were frozen in liquid nitrogen. Cell lysis and metabolite extraction were performed as described previously (Zech et al., 2009; Dannheim et al., 2017b). One ml of the polar phase was dried in a vacuum concentrator and stored at -80°C prior to analysis. Extracellular samples were prepared as described previously (Neumann-Schaal et al., 2015). Volatile and non-volatile compounds in the culture supernatants and cell free extracts were analyzed via GC/MS as described earlier (Neumann-Schaal et al., 2015). Raw data obtained from GC/MS measurements were processed by applying version 2.2N-2013-01-15 of the in-house developed software MetaboliteDetector (Hiller et al., 2009). The peak identification was performed in a non-targeted manner with a combined compound library. After processing, non-biological peaks and artifacts were eliminated with the aid of blanks. Peak areas were normalized to the corresponding internal standards (o-cresol or ribitol) and derivatives were summarized. Significant changes in metabolite levels were calculated by non-parametric Wilcoxon–Mann–Whitney test (Mann and Whitney, 1947) using Benjamini–Hochberg correction (Benjamini and Hochberg, 1995) to control the false discovery rate. Metabolome data are summarized in Supplementary Table S6.

### HPLC/MS-Based Analysis of Coenzyme A-Derivatives

Coenzyme A (CoA)-esters were isolated by cell breakage using a Precellys 24 homogenizer (Peqlab, Erlangen, Germany) at -10°C. The procedure included three cycles of homogenization (6,800 rpm, 30 s with equivalent breaks). The lysate was transferred to 10 ml of ice-cold ammonium acetate (25 mM, pH 6) and centrifuged (5 min at 10,000 *g*, 4°C). CoA-derivatives were extracted on a Strata XL-AW solid phase extraction column (Phenomenex, Aschaffenburg, Germany) as described previously (Wolf et al., 2016). CoA-derivatives were analyzed on a Dionex ultimate 3000 system (Thermo Scientific Inc., Darmstadt, Germany) coupled to a Bruker MicroTOF QII mass spectrometer (Bruker Daltonik GmbH, Karlsruhe, Germany) equipped with an electrospray ionization interface. The separation and detection was performed as described previously (Peyraud et al., 2009; Wolf et al., 2016). Raw data were processed using the XCMS package (Smith et al., 2006; Benton et al., 2008; Tautenhahn et al., 2008) for R (version 3.0.3) as described previously (Wolf et al., 2016). Significant changes in metabolite levels were calculated by non-parametric Wilcoxon-Mann-Whitney test (Mann and Whitney, 1947) using Benjamini–Hochberg correction (Benjamini and Hochberg, 1995) to control the false discovery rate. Metabolome data are summarized in the Supplementary Table S6.

### Bioinformatics

#### *De novo* Motif Search

Motif search was performed with the standalone version of MEME (Bailey et al., 2009) on the promoter sequences [-250,0] of 66 genes known to be differentially regulated by Fur and involved in the iron metabolism. MEME was run with option “-anr” and without any restrictions on the motif width. Motif presence was confirmed in 11 out of the 66 promoters.

#### Genome-Wide Motif Scan

We performed genome-wide motif search in the promoters [-250,0] of *C. difficile* using the *de novo* obtained position weight matrix (PWM). The standalone version of the MAST tool available in the MEME package was run with option “-norc” (search only the forward strand) once with default other parameters and once with maximal motif hit *P*-value of 5.10(-5).

## Results

### Construction of a *C. difficile fur* Mutant and Definition of High and Low Iron Growth Conditions

The overarching aim of this study was identification and characterization of the Fur regulon at the transcriptional, translational, metabolomics and the phenotypic level. For this purpose a *fur* mutant was constructed using the ClosTron technology (Heap et al., 2007, 2009, 2010). The *fur* gene was identified and characterized before by Ho and Ellermeier (2015) and partially by Dubois et al. (2016). Similar to their approaches, a stabile insertional mutation in open reading frame CDIF630_01441 of the laboratory strain *C. difficile* 630Δ*erm* (Hussain et al., 2005) was generated. The ClosTron system uses a group II intron to insert an erythromycin resistance cassette into the target gene. *C. difficile* 630Δ*erm*, an erythromycin-sensitive derivative of *C. difficile* strain 630, was used as the parental strain and is further referred to as wild type. The insertional mutant was confirmed by PCR analysis (Supplementary Figure S1). The growth behavior of the wild type and the constructed *fur* mutant in logarithmic growth phase was almost identical when tested in the complex Brain-Heart-Infusion (BHI) medium independent of the addition of iron (Supplementary Figure S2). However, the stationary phase was entered earlier by the *fur* mutant. Similar observations have been made for the *fur* mutant grown in complex TY medium before (Ho and Ellermeier, 2015). A different growth behavior was observed in Clostridium Minimal Medium (CDMM). Here we tested high (15 mM) and low (0.2 mM) concentrations of iron, in this case iron sulfate. For this purpose a commercial analytical laboratory (Currenta Analytik, Leverkusen, Germany) investigated the CDMM used throughout this investigation with Inductively-Coupled-Plasma Mass Spectrometry (ICP-MS) for its iron content. Highly reproducible, 0.2 mM iron were determined for the medium and used as low iron conditions. For defining high iron conditions CDMM was titrated with increasing amounts of iron and *C. difficile* wild type growth stimulation was determined. When the point of no further growth stimulation was reached 9.2 mM iron were measured by ICP-MS in CDMM.

**FIGURE 1:**
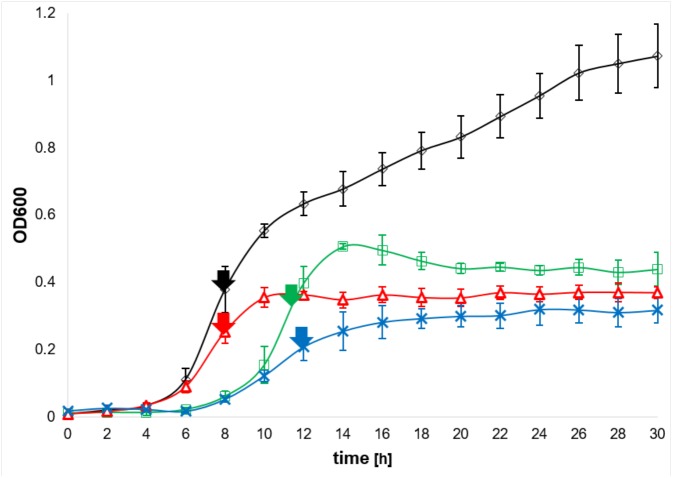
Growth of wild type and *fur* mutant growth at low and high iron concentration. Growth curves of *Clostridioides difficile* wild type and the corresponding *fur* mutant in CDM medium with 15 μM iron sulfate (high iron, black for wild type and green symbols for the *fur* mutant) or 0.2 μM (low iron, red for wild type and blue symbols for the *fur* mutant) are shown. Growth was monitored every two in at least five independent cultivations. Arrows indicate time points of sampling for the systems biology (Omics) approaches. Standard deviations are indicated.

We explicitly circumvented the utilization of the chelator 2,2′-dipyridyl (DPP) to achieve low/no iron conditions. Cernat and Scott failed after DPP treatment of *C. difficile* to recover the bacterial growth by the addition of alternative iron sources including lactoferrin, transferrin, hemoprotein, and heme (Cernat and Scott, 2012). A high-throughput small molecule screen identified DPP as one of the most potent inhibitors of *C. difficile* growth, even in a mouse model (Katzianer et al., 2014). Latter indicated the importance of iron for *C. difficile* growth, but also showed the detrimental effects of DPP treatment. Nevertheless, DPP remains an useful and often used tool to achieve complete iron depletion. To our understanding *C. difficile* does not encounter completely iron free conditions in its environment, thus, we compared high (15 μM) and low (0.2 μM) iron conditions in all experiments of this study. When growth of the wild type and the *fur* mutant was compared under both iron concentrations, both strains revealed significant reduced growth under iron limited conditions (Figure 1). Complementation of the *fur* mutant with a plasmid encoded *fur* restored growth to almost wild type conditions (Supplementary Figure S3). Furthermore, the *fur* mutant grew much slower and to lower terminal densities compared to the wild type strain. Obviously, Fur is required for optimal growth under high and low iron conditions (Figure 1).

Next, different iron sources were analyzed for the ability to restore iron limited growth of the wild type and the *fur* mutant (Supplementary Figure S4). Addition of 15 mM iron citrate or iron (II) chloride induced a growth behavior of *C. difficile* similar to that observed for the addition of iron sulfate (compare Figure 1 and Supplementary Figures S4A,B,D). Addition of 10 μM hemin, or 10 μg/ml transferrin did not significantly improve wild type growth, but slightly enhanced the growth of the *fur* mutant. Substitution with 10 μg/ml ferritin clearly improved the growth of both strains (compare Figure 1 and Supplementary Figures 4C,E,F). Overall, the basic difference in the growth behaviors of the wild type and the mutant strain remained similar under various tested iron conditions, i.e., the various iron sources did not compensate for the loss of Fur in the mutant strain. Obviously, additional functions besides iron regulation are executed by Fur in *C. difficile*.

### Transcriptome, Proteome and Metabolome of Wild Type and the *fur* Mutant of *C. difficile* Grown at Iron-Limiting and Iron-Saturated Conditions

We aimed at a multi-level, holistic view on iron-regulation in *C. difficile* and the contribution of Fur to these processes. To analyze environmental iron conditions close to the gut habitat, we refrained from DPP treatment of the cultures, rather we compared samples taken from low, iron limiting growth conditions (0.2 μM) with samples of iron saturated (15 μM) growth conditions. The transcriptome (RNA-Seq), cytoplasmic proteome, metabolome, and exo-metabolome of wild type and the *fur* mutant grown under both conditions were compared. Samples were taken in the exponential growth phase as indicated by arrows in Figure 1. This approach enabled us to functionally identify iron regulated processes at the transcriptional and proteomic level, and to observe their metabolic consequences. Furthermore, the inhibitory and promoting activities of Fur became visible. Certain phenotypes were further investigated using electron microscopy and growth experiments.

**FIGURE 2:**
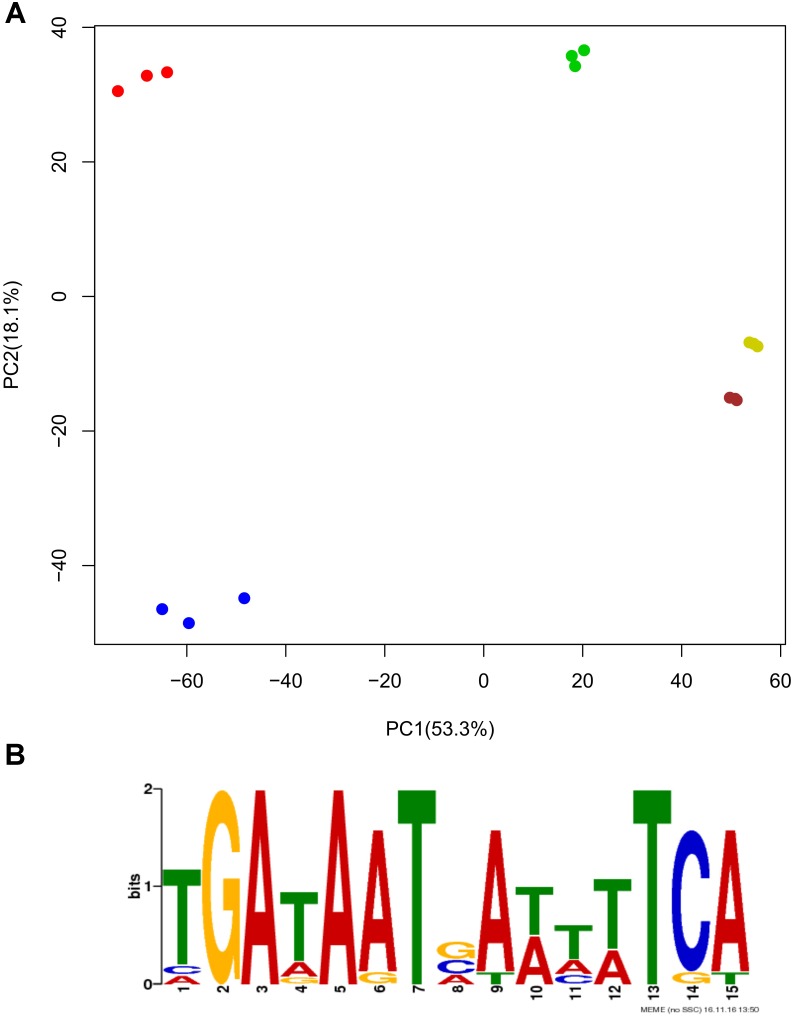
**(A)** Principle component analysis (PCA) of transcriptome data from previously published datasets and this study concerning Fur regulated adaptation to low/no iron conditions and sequence logo for the deduced Fur binding site. Data are shown as triplicates recorded after growth in iron rich medium [red, wild type – this study, yellow, same – from (Ho and Ellermeier, 2015), green, same from (Hastie et al., 2018), blue – *fur* mutant, this study, brown – from (Ho and Ellermeier, 2015)]. **(B)** Position weight matrices deduced Fur binding site sequence logo is shown on the bottom (see also Supplementary Table S7).

The RNA-Seq approach identified 3,156 individual transcripts. First, we compared the 4 different transcriptomes (wild type low/high iron, *fur* mutant low/high iron) by principal component analyses (Supplementary Figure S5). Interestingly, biological triplicates from wild type/low iron, *fur*/low iron, and *fur*/high iron showed a certain degree of overlap, while triplicates for wild type/high iron clustered very distinct. As Fur usually acts as a transcriptional repressor at high iron concentration, global transcriptional changes due to high iron availability were mostly effected by the presence of active Fur. The terms “induced” and “repressed” were used for enriched or depleted RNAs throughout the paper. We are fully aware of the fact that comparative RNA-Seq shows changes in RNA abundancies, which might not always correlate with changes in gene expression. Using a log2 fold change of 1 in transcript abundance (*p*-value of 0.05) as cutoff, 243 genes were found up- and 303 genes down-regulated in response to iron limitation (Supplementary Tables S1–S3). Comparing wild type and the *fur* mutant at high iron 369 genes were found up- and 268 genes found down-regulated (Table 1). In order to visualize the differences of the currently available transcriptome wild type (Ho and Ellermeier, 2015; Hastie et al., 2018) the principal component analysis was employed for all available transcriptome data of *C. difficile* wild type versus *fur* mutant at high iron growth conditions. Results are summarized in Supplementary Tables S1–S3. We were aware of the fact that highly different transcriptome methods (RNA-Seq versus DNA array) and different low/no iron condition (with and without DPP) were compared. Clear cut differences became visible (Figure 2). The DNA array data of the wild type obtained in the presence of high iron and low/no iron (Hastie et al., 2018) cluster together, nevertheless, with some distance. The DNA array data for the *fur* mutant obtained at high iron conditions (Ho and Ellermeier, 2015) cluster separate from the RNA-Seq data, however, with the wild type data oriented toward the RNA-Seq wild type data and the *fur* mutant data toward the RNA-Seq *fur* mutant data (Figure 2).

Analyzing iron limitation in *C. difficile* with a proteomics approach, a total of 1,639 proteins were identified. A recent investigation of 8 *C. difficile* proteome yielded 662 quantifiable common proteins (Dresler et al., 2017). Using a cutoff at a log2 fold of 1 (*p*-value of 0.05) 85 proteins were found down- and 61 up-regulated in response to iron limitation. A total of 170 proteins were not found (OFF) and 85 solely found (ON) under iron limiting conditions (Supplementary Table S5). For the wild type versus *fur* mutant comparison 1,682 proteins were analyzed. Using the same cutoff 86 proteins were found depleted and 122 enriched in the *fur* mutant compared to wild type, both grown at high iron conditions. A total of 152 proteins were not detected (OFF) and 128 proteins solely identified (ON) in the *fur* mutant (Supplementary Table S4). Comparing transcriptome and proteome data, interesting differences were observed, most likely reflecting the delay of the response of the proteome compared to the transcriptome at the analyzed time point (Table 1). These differences will be described and discussed in the context of the various regulated processes below. Furthermore, a significant degree of similarity was observed for the Omics data for high versus low iron and the wild type versus *fur* mutant at high iron conditions, indicating that major adaptations were controlled directly or indirectly by Fur (Supplementary Table S4).

Combined GC/MS- and LC/MS-based metabolome approaches were employed for the analyses of intracellular metabolites including CoA-esters and for the elucidation of the metabolic composition of the growth medium and corresponding volatiles. Overall, we identified 113 intracellular metabolites including 23 coenzyme A-esters. Extracellularly, 45 metabolites were identified. Using a fold change cutoff of 1.5 and at an adjusted *p*-value of 0.05, 52 metabolites were found in higher concentration and 13 in lower concentration under low iron conditions (Supplementary Table S5). For the wild type versus *fur* mutant comparison using the same cutoff 29 metabolites were found more and 31 less abundant in the *fur* mutant compared to wild type when both were grown at high iron conditions. Overall, the most abundant identified metabolites were dominated by amino acids and their products (5-aminovalerate, glutamate, valine, isoglutamate, leucine, lysine, alanine and more) followed by diverse coenzyme A-esters, cofactors and polyamines (spermine, spermidine). As typically observed for *C. difficile*, only a few sugars and activated sugars (glucose, glucose-6-phosphate and fructose-1,6-bisphosphate) or intermediates of the central carbon metabolism (2-oxoglutarate) were under the highly abundant metabolites (Supplementary Table S6).

Finally, a bioinformatics approach for the definition of the Fur regulon was taken. Fur binding sites upstream of Fur-regulated genes in *C. difficile* were combined to define a position weight matric using the MEME motif search tool version 4.11.2. A consensus binding site of TGATAATVAWHWTCA was deduced (Figure 2). Overall, 161 potential strand-specific Fur binding sites were identified up to 250 bp upstream of 147 coding genes/operons. Approximately 20 of these binding sites were found upstream of genes involved in the regulation of the major adaptations processes to low iron condition in *C. difficile* (Supplementary Table S7).

### Fur-Mediated Iron Regulation of Metal Uptake Systems

**FIGURE 3:**
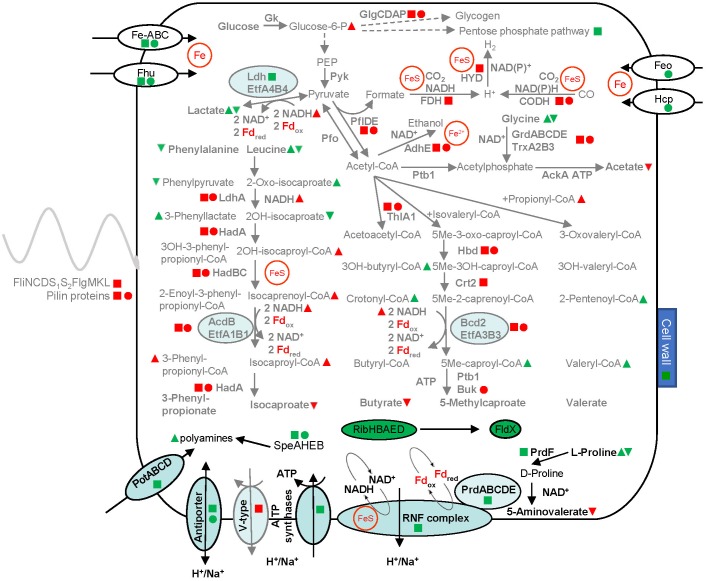
Overview of the overall adaptation strategies of *C. difficile* to low iron conditions. Transcriptome (RNA-Seq), proteome and metabolome data were integrated into a general adaptation strategy model. Shown are enzymes (bold) and metabolites (non-bold), generally upregulated pathways are shown in black, while downregulated pathways are labeled in gray. The changes in abundance of the corresponding mRNAs, proteins and metabolites between low iron and high iron and/or a comparison between the *fur* mutant and the wild type strain are indicated in the following code: Transcriptome (RNA–Seq) data are shown as squares, proteome data as circles, metabolome data as triangles (cytoplasmic metabolome, peak up, exo-metabolome, peak down). Green indicates higher abundance and red indicates a reduction of the cellular abundances of the corresponding molecules. Filled symbols indicate the same effect in both conditions (high versus low iron and wild type versus fur mutant), open symbols indicate the effect in only one condition, blue symbols indicate contrary effects. Cut off values were a log_2_ fold change of 2 for transcriptome and proteome and a fold change of 1.5 for metabolic data. Iron-dependent reactions are labeled by brown circles and letters (Fd, ferredoxin; FeS, iron sulfur clusters, Fe^2+^). Fe-ABC, YclNOPQ; OH, hydroxy-group; CoA, coenzyme A; Me, methyl group. For details, see Table 1, and the Supplementary Tables.

As expected various iron and other metal uptake systems encoded by *fpi*, *fhu*, *zupT* and the sulfonate transporters encoded by the *ssu* operon (CDIF630erm_03273–03276) were found more abundant by low iron conditions at the transcriptome and proteome level (Table 1). This response was indirectly mediated by Fur, since a conserved Fur binding site was not detected upstream of the *ssu* operon. This is in agreement with previously published transcriptome analyses induced (Ho and Ellermeier, 2015; Hastie et al., 2018). In the previous two transcriptome analyses using DPP-treated bacteria as iron depleted condition, the ferrous iron uptake transporter genes *feoA1* (CDIF630erm_01641 – 01642) were also found clearly induced (Ho and Ellermeier, 2015; Hastie et al., 2018). Moreover, low induction by iron depletion was observed for *feoA5*/*feoB3* (CDIF630erm_03573 – 03574) and *feoA4* (CDIF630erm_01939). None of the FeoA type systems were found more abundant at the transcriptome level in our approach with 0.2 μM iron as low iron conditions. However, the proteome data revealed that FeoAB system encoded by CDIF630erm_01641 – 01643 was induced at low iron condition in a Fur-dependent manner (Table 1). Moreover, the significant differences in the observed fold changes in gene induction between DPP-treated cells (up to 730-fold with the DNA array, over 100-fold for the RNA-seq) and 0.2 μM iron grown cells (around 5-fold) might further explain some of these observations. Possibly, at an iron concentration of 0.2 μM the necessary threshold of iron depletion for the Feo-type systems was not reached. Alternatively, *feo* gene regulation by low iron with Fur was of transient nature and finished at the time point of sampling. Possibly, the adaptation at this certain time-point was only visible at the proteome level (summarized in Figure 3). Many of the operons/genes (CDIF630erm_01824, CDIF630erm_01827, CDIF630erm_03145, CDIF630erm_03146, CDIF630erm_01641, CDIF630erm_01643, CDIF630erm_01939) involved in iron uptake contain potential Fur binding sites in their upstream region, indicating direct Fur control. Nevertheless, similar results were obtained for the currently available three transcriptome analyses (Ho and Ellermeier, 2015; Hastie et al., 2018). Overall, this response was clearly coordinated directly by Fur, indicated by the multiple potential binding sites (Supplementary Table S7). Some of them were already confirmed by DNA binding studies before (Ho and Ellermeier, 2015).

With standard Western diet the iron concentrations in the gut is about 100 mg Fe/g wet weight feces (Pizarro et al., 1987; Lund et al., 1999). However, due to the rising pH in the duodenum and the small intestine solubility of ferric iron decreases and favors the oxidation to ferrous iron in the presence of oxygen. Furthermore, ascorbic acid and citric acid chelate iron and make it available to the microbiome and host. On the other side polyphenols like tannins and catechols from tea or coffee as well phytate from cereals tightly bind iron. Consequently, the amount of iron in the colon lumen that is readily available to bacteria is difficult to estimate (Kortman et al., 2014). The large amounts of different siderophores found in the feces indicate strongly limited access to ferric iron for the gut microbiome (Kortman et al., 2014). In summary, there is always some iron around in the gut. However, the actual iron concentrations might vary with respect to nutrition. Consequently, high and low affinity iron uptake systems are advised. Most likely the differences observed between the proteome and transcriptome data regarding induction of iron-uptake systems of this study as well as the difference to the two transcriptome analyses performed before might be caused by a time-resolved response to iron limitation.

Certain systems found still enhanced at both the RNA and protein level (*yclP*, *ssuA2, ssuB2*, CDIF630erm_01231), while other were already formed and the increased abundance became only visible at the protein level (*feoA*, CDIF630erm_01642; *feoB1* CDIF630erm_01643). A co-regulation of the sulfur (*ssu* operon) and iron metabolism becomes obvious and was observed before (Dubois et al., 2016). This might be explained by the often sulfur-mediated iron coordination in enzymes of *C. difficile* (see Supplementary Table S8). One of the strongest induced operons at no/low iron conditions in all three transcriptome analyses (Ho and Ellermeier, 2015; Hastie et al., 2018) was the one encoding the catecholate siderophore import system YclNOPQ (CDIF630erm_001824 – 01827). This represents a high affinity iron import system induced at low iron conditions and in the fur mutant which allows uptake of iron at low bioavailability.

The precursor of many catechol siderophores is spermidine (Datta and Chakrabartty, 2014). Interestingly, the spermidine biosynthesis genes *speAHEB* (CDIF630erm_01008 – 01022) and spermidine/putrescine transporter genes *potABCD* (CDIF630erm_01160 – 01163) were significantly induced on the transcriptome (*spe*, Hastie et al., 2018 and *pot*) and the proteome (only *spe*) level (Table 1 and Figure 3). In agreement, significantly increased levels of spermidine, spermine and putrescine were detected in the metabolome of iron limited *C. difficile* cells (Supplementary Table S6). Already in the nineties an increase in polyamines in bacterial cell grown under iron-limited conditions was studied (Bergeron and Weimar, 1991). A similar close relationship between intracellular iron and polyamine content was described for cancer cells (Bae et al., 2018; Lane et al., 2018). Interestingly, siderophores like petrobactin (Lee et al., 2007), alcaligin (Challis, 2005) are formed from polyamines. Others like vibriobactin and vulnibactin contain polyamine backbones (Shah and Swiatlo, 2008; Bergeron et al., 2011). However, the protective function of polyamines during stress situation and their importance for the infection process of many bacteria have been widely described (Shah and Swiatlo, 2008).

### The *fur* Mutant Lost Most of Its Flagella and Motility

**FIGURE 4:**
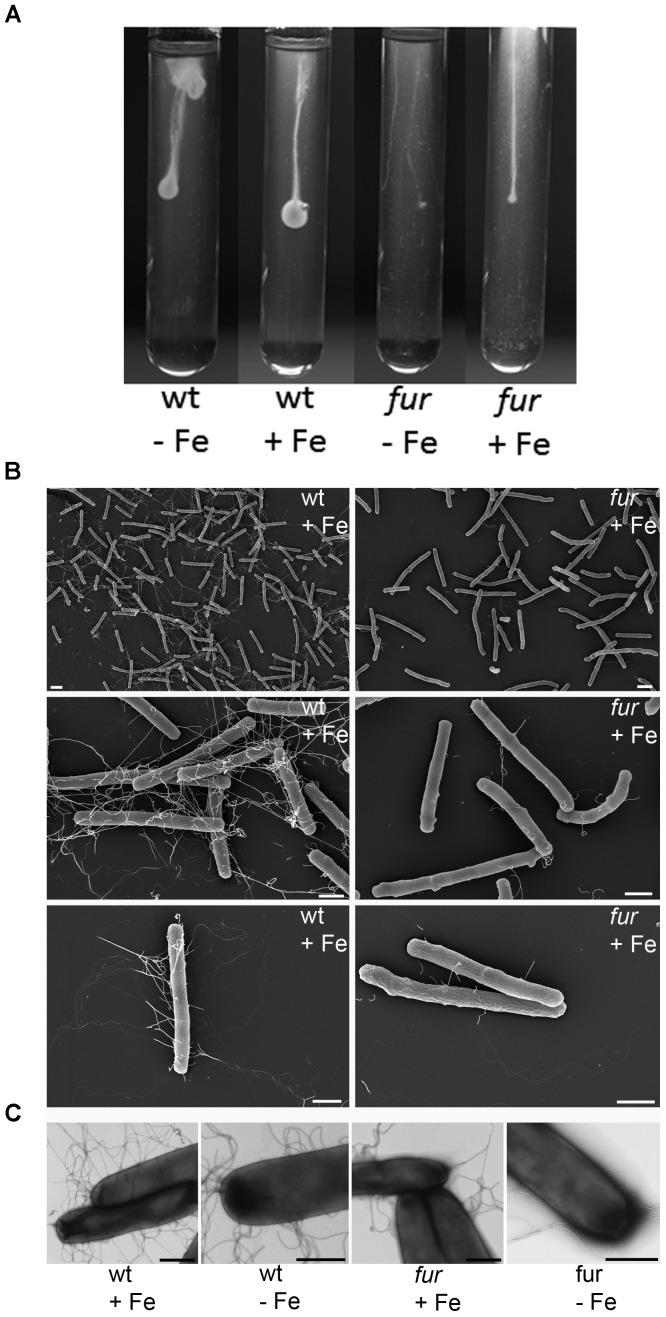
Scanning/transmission electron microscopic images and motility assays of *C. difficile* wild type and the corresponding *fur* mutant. **(A)** CDMM agar filled glass tubes containing 0.2 μM (-Fe) and 15 μM (+Fe) iron sulfate were inoculated with wild type (wt) and the corresponding fur mutant strain and incubated anaerobically for 24 h. Scanning electron microscopy picture of *C. difficile* (**B**, right panel) and the *fur* (mutant **B**, left panel) grown in CDMM containing 0.2 μM (-Fe) and 15 μM (+Fe) iron sulfate are shown. Negative staining **(C)** also depicts less flagellation of the *fur* mutant and no detectable other appendage-like structures on the surface of *C. difficile* like pili or fimbriae. Bars represent 2 μm in **(B)**, top 2 images and 1 μm in all other images.

Comparative inspection of the wild type and *fur* mutant *C. difficile* strains using scanning electron microscopy revealed obvious differences with regard to the presence of flagella. Scanning electron microscopy revealed a significant loss of flagella in the *fur* mutant compared to the wild type *C. difficile* (Figure 4B). Negative staining (Figure 4C) also depicted less flagellation of the *fur* mutant and no detectable other appendage-like structures on the surface like pili or fimbriae. Motility assay revealed in agreement with the electron microscopy analyses, that the *fur* mutant was highly impaired in motility (Figure 4A). Interestingly, the two flagella operons were also subject to Fur-mediated gene regulation, one (CDIF630erm_00375 – 00395) was found Fur-repressed, while the other (CDIF630erm_00348 - 00361) was identified as Fur-induced. Interestingly, latter operon contained a Fur box upstream of CDIF630erm_00348. The missing Fur-dependent induction of this operon might have caused the observed phenotype. The proteome data partially confirmed this assumption. Most likely, additional unknown factors are required. Remarkably, the gene for the pleiotropic regulator SinR (CDIF630erm_02447) was found overexpressed under iron limiting conditions. One function of the SinR regulator in *C. difficile* is the induction of flagella formation and motility via the control of c-di-GMP levels (Girinathan et al., 2018). Similarly, proline iminopeptidase (CDIF630erm_02215), catalyzing the removal of N-terminal proline residues from peptides, was described to be involved in *Xanthomonas campestris* motility via influencing c-di-GMP levels (Khan et al., 2018). The corresponding *plp* gene and a TetR family transcriptional regulator were found induced under low iron conditions. Pili gene transcription (CDIF630erm_03817 – 03826) was found reduced in the *fur* mutant (Table 1). This is in agreement with the electron microscopy inspection of the *fur* mutant (Figure 4C). Due to their extracellular location only one pilus protein was detected by the proteomics approach, but as expected solely in the wild type strain (OFF). Due to a missing potential Fur box upstream the *pil* operon the observed regulation might be of indirect nature.

### Low Iron Conditions Induce Major Re-Arrangements of the Energy Metabolism Partially Regulated by Fur

The basic principles of energy generation of *Clostridia* differs significantly from those of eukaryotes or other prokaryotes. Some of these bacteria mainly generate their energy in form of amino acid fermentation using two coupled reactions previously called Stickland reaction (Stickland, 1934). ATP is formed at the substrate level and using a proton/sodium gradient at the membrane. In principle, during the first oxidative part of the reaction, the first amino acid gets deaminated to form an a-keto acid with the concurrent transfer of the electrons to a carrier like NAD^+^. Subsequently, the decarboxylation of the a-keto acid is linked with the formation of a coenzyme A ester, which in turn is converted into an acyl-phosphate. The final transfer of the activated phosphate residue to ADP yields ATP. In the reductive part of the pathway the second amino acid gets reduced by the formed electrons and deaminated. Sometimes this process is again coupled to ATP generation (Durre, 2014). During the analysis of butyrate formation in *C. difficile* the enzyme butyryl-CoA dehydrogenase (Bcd) was identified as an electron bifurcating stable complex with the flavoproteins EtfA and EtfB (Aboulnaga et al., 2013). The complex oxidizes NADH and transfers two electrons to the first flavin (β-flavin), which bifurcates one electron to ferredoxin and one electron to a second flavin (α-flavin). After two such rounds the completely reduced a-flavin transfers two electrons further to the third flavin (s-flavin) of the complex, which finally reduces crotonyl-CoA to butyryl-CoA (Chowdhury et al., 2014; Demmer et al., 2017). Most importantly, formed reduced ferredoxins are the substrate of the membrane spanning ferredoxin-NAD^+^ reductase complex (Rnf) which couples the electron transfer from the ferredoxin to NAD^+^ with the generation of a proton or sodium gradient at the membrane (Biegel and Muller, 2010; Tremblay et al., 2012; Mock et al., 2015; Chowdhury et al., 2016). The generated sodium gradient drives ATP generation via a sodium-dependent ATPase (Buckel and Thauer, 2013; Buckel and Thauer, 2018).

*Clostridioides difficile* possesses three different EtfAB systems. The first is encoded downstream the *bcd2* gene encoding butyryl-CoA dehydrogenase (CDIF630erm_01194 – 01199), the second (CDIF630erm_01319 – 01320) in an operon with lactate racemase (LarA, CDIF630erm_01318) and a lactate dehydrogenase (CDIF630erm_01321) and the third downstream of *acdB* encoding a short chain acyl-CoA dehydrogenase involved in the conversion of 2-enoyl-3-phenylpropionyl-CoA/isocaprenoyl-CoA into 3-phenylpropionyl-CoA/isocaproyl-CoA during phenylalanine/leucine fermentation with formation of 3-phenylpropionate/isocaproate (Elsden and Hilton, 1978; Britz and Wilkinson, 1982; Kim et al., 2006). The selenoprotein D-proline reductase (PrdABCDE) catalyzes the reductive ring cleavage of D-proline to form 5-aminovalerate. As typical Stickland reaction it is coupled to the oxidation of other amino acids, but also formate can serve as electron donor (Kabisch et al., 1999). First proline racemase (PrdF) converts L-proline into D-proline (Wu and Hurdle, 2014). Already in the eighties it was shown that proline reduction is coupled to proton motive force generation (Lovitt et al., 1986). However, the enzyme complex does not reduce ferredoxin and was proposed to directly interact with the membrane-localized, proton/sodium pumping Rnf complex (Jackson et al., 2006). Glycine reductase (GrdABCDE) catalyzes the reductive deamination of glycine to form acetylphosphate and ammonia with the oxidation of thioredoxin (TrxA2, TrxB3) (Andreesen, 2004). The influence of iron on the fermentative metabolism of *Clostridium acetobutylicum* was already described in the eighties (Bahl et al., 1986).

At low iron conditions the ferredoxin-dependent pathways of phenylalanine, leucine (*hadAIBCB*, *etfBA*, CDIF630erm_00523 – 00529), glycine degradation (g*rdDCBAE*,*trxBA* (CDIF630erm_02587 – 02597) and butyrate fermentation (CDIF630erm_01194 – 01199) with the formation of 3-phenylpropionate, isocaproate, butyrate, 5-methylcaproate, valerate and acetate (CDIF630erm_01194 – 01199, CDIF630erm_02577 – 02583) were significantly reduced, while the proline reductase (*prdFEDBARC*, CDIF630erm_03533 – 03544) and Rnf complex encoding operons (*rnfCDGEAB*, CDIF630erm_01284 – 01289) were induced in *C. difficile* (Table 1). Correspondingly, the highest increase in metabolite concentration was observed for 5-aminovalerate, the product of up-regulated proline utilization, when the lower biomass responsible for its production is taken into account. In agreement, the substrates of the reduced pathways phenylalanine, leucine, glycine and some initial intermediates (phenylpyruvate, 2-oxo-isocaproate, 3-hydroxybutyryl-CoA, crotonyl-CoA) were found accumulated. At the same time some end product like isocaproate and butyrate were found reduced (Figure 3).

Moreover, the synthesis of the flavodoxin (FldX, CDIF630erm_02217) and of enzymes of riboflavin biosynthesis (*ribHBAED*, CDIF630erm_01882 – 01885) was found significantly enhanced. Interestingly, flavodoxins can replace ferredoxins as electron donors for the proton/sodium ion pumping ferredoxin-NAD^+^ reductase (Rnf) (Chowdhury et al., 2016). Consequently, one explanation for the increased formation of flavodoxins is the replacement of iron-containing ferredoxin as electron donors at the Rnf-complex. The EtfAB (CDIF630erm_01319 - 01321) containing system with nickel-dependent lactate racemase (CDIF630erm_01318) was found induced under low iron conditions (Weghoff et al., 2015). Possibly, the yet unknown function contributes to the overall change or the system uses flavodoxin as natural electron acceptors. Interestingly, a change in ATPase also accompanied the switch from high to low iron conditions. Under low iron conditions, where the directly Rnf complex-coupled proline reductase was found enhanced, an F_0_F_1_-type, sodium-dependent ATP forming ATPase (*atpCDGAHFEB*, CDIF630erm_03778 – 03785) was found induced. Under high iron conditions Fur-induces the formation of a V-type, mostly proton-pumping, ATP-consuming ATPase (*atpDBAFCEKI*, CDIF630erm_03237 – 03245) was preferentially produced. Promoter sequences upstream of the latter genes contained a potential Fur biding site. Clearly, under low iron conditions *C. difficile* significantly reduced the formation of most iron-requiring, ferredoxin-dependent processes including phenylalanine/leucine utilization via AcdB/EtfA1B1 and butyrate/caproate/valerate formation via Bcd2, EtfA3B3 (Table 1 and Figure 3). Only lactate formation via Ldh/EtfA4B4 was found. Finally, the transcripts for an F_0_F_1_-type, sodium-dependent ATP forming ATPase (*atpCDGAHFEB*, CDIF630erm_03778 – 03785) were more abundant, while the formation of a V-type, mostly proton-pumping, ATP-consuming ATPase (*atpDBAFCEKI*, CDIF630erm_03237 – 03245) was reduced. Obviously, the ferredoxin-independent process of Rnf-complex coupled proline utilization was found enhanced and with it the formation of an ATP forming proton/sodium–driven ATPase. The oligopeptide transporter OppBCAD (CDIF630erm_0972 - 0975) was found reduced.

Looking at the identified potential Fur binding sites, most of the observed changes are not directly regulated by Fur. Two open readings frames upstream of the *had* operon and the hydroxybutyrate metabolizing enzymes encoding operon possessed potential Fur binding sites (Table 1). Additionally, the flavodoxin gene *fldX* contained a Fur-box upstream its coding region. Perhaps, known regulators including PrdR, CodY, CcpA or Rex are involved in the detection of the drastic physiological changes accompanying the outlined adaptation process (Bouillaut et al., 2015). Currently, the relationship between membrane protein function and lipid composition becomes true. In this context, the major operon of fatty acid biosynthesis (*fapR*, *plsX*, f*abHKDG*, *acpP*, *fabF*, CDIF630erm_01326 – 01333) was up-regulated at the transcriptional level during iron limiting conditions (Table 1). Obviously, a re-structuring of the membrane is required for the overall adaptation of multiple membrane associated metabolic processes.

### Iron Requiring Metabolic Processes of the Central Metabolism and of CO Oxidation Are Significantly Downregulated at Low Iron Conditions

*Clostridioides difficile* is utilizing pyruvate via the radical enzyme pyruvate formate-lyase, which forms in the presence of coenzyme A acetyl-CoA and formate (Figure 3). Formate gets subsequently oxidized by the formate dehydrogenase H to CO_2_ and protons. The [NiFe] Hydrogenase Hyd reduces protons to molecular hydrogen (Shafaat et al., 2013; Pinske and Sawers, 2016). Pyruvate formate-lyase (PflD) requires an [4Fe-4S] cluster containing activating enzyme (PflC, PflD1) for the formation of the catalytic glycyl radical (Crain and Broderick, 2014). Formate lyase H (FdhF) is described as a MoCo-containing selenoprotein with a single [4Fe-4S] cluster (Pinske and Sawers, 2016). FdhD is a sulfurtransferase which transfers the sulfur residing on the desulfurase IscS to FdhF (Thome et al., 2012). The [NiFe] Hydrogenase (HydN1AN2) contains 3 different iron-sulfur clusters and heme (Shafaat et al., 2013; Pinske and Sawers, 2016). The whole array of Fe-containing enzymes and their activators were found strictly down-regulated under low iron conditions (Supplementary Table S8). The utilization of glucose via pyruvate-formate lyase (PflD, CDIF630erm_03582 – 03583), with formate dehydrogenase and a hydrogenase (Hyd, Fdh, CDIF630erm_03614 – 03619) was downregulated mainly at the transcript level at low iron condition (Table 1 and Figure 3). Consequently, the whole flux toward formate and hydrogen was significantly blocked at low iron conditions, glucose and pyruvate accumulated (Supplementary Table S5). The overall flux through the glycolysis seemed to be reduced since also glucose accumulated 2.17-fold (Figure 3). In agreement the synthesis of the enzyme for glycogen formation from glucose (GlyCDAP) was also diminished by 3.5-fold. Again, an iron-requiring metabolic pathway was shut down at low iron conditions. Fur-dependent regulation might be mediated via a potential Fur-box found upstream the hydrogenase gene *hydN2* and the ATPase gene *atpA*. CO dehydrogenase (CooSC, CDIF630erm_00832 – 00833) formation was found reduced at the transcriptional and proteomic level. Carbon monoxide dehydrogenase CooSF contains 5 [Fe-S] cluster and catalyzes the oxidation of CO using water with the formation of CO_2_ and hydrogen (Dobbek et al., 2001). Like the hydrogen utilizing hydrogenase, carbon monoxide dehydrogenase was down-regulated at the transcriptional and proteomic level.

### Cell Wall Restructuring and the Protection Against Antibiotics and CAMPs

Obviously, the low iron stress was counteracted via increased resistance to external attacks. Firstly, the transcription of the *dtl* operon (*dtlCBAD*, CDIF630erm_03118 - 03122) involved in the resistance to the collection antimicrobial peptides (CAMP) was enhanced during low iron conditions (McBride and Sonenshein, 2011). The enzymatic system encoded by the corresponding genes is responsible for the D-alanylation of lipoteichoic acids. The D-alanine-poly(phosphoriboto) ligase DltA ligates D-alanine to the carrier protein DltC. Aided by DltB, DltC transferres the D-alanine further to undecaprenyl phosphate and transverses to the membranes. Finally, the D-alanine transferases DltD is involved in the final release of the lipoteichoic acids outside the cell. Similarly, the so called vancomycin resistance gene cluster (*vanGYTG*, CDIF630erm_01803 – 01805) was found induced at the transcriptional level. The encoded proteins VanG (D-Ala:D-Ser ligase), VanXY (D,D-depeptidase), and VanT (D-Ser racemase) acting on the peptidoglycan, were found all functional in *C. difficile* before, however, confer only low resistance to vancomycin (Ammam et al., 2013; Peltier et al., 2013).

**FIGURE 5:**
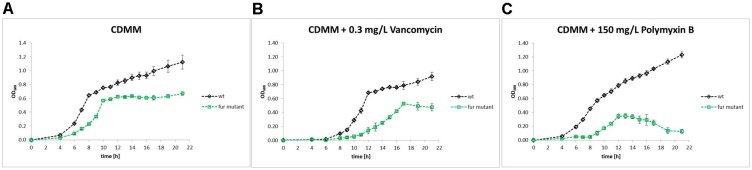
Growth of wild type and *fur* mutant growth at high iron concentration in the presence of vancomycin and polymyxin B. Growth curves of *C. difficile* wild type and the corresponding fur mutant in CDM medium with 15 μM iron sulfate, without **(A)** and with the addition of 0.3 mg vancomycin/l **(B)** and 150 mg polymyxin B/l **(C)** are shown. Black symbols are used for the wild type and green symbols for the fur mutant. Growth was monitored every 2 h in at least five independent cultivations. Standard deviations are indicate.

We challenged the wild type and the *fur* mutant with below MIC_50_ amounts of vancomycin and the CAMP polymyxin B as determined before (McBride and Sonenshein, 2011; Ammam et al., 2013). In the presence of 0.3 mg vancomycin/l a delayed growth of the wild type and the fur mutant with an visibly increased sensitivity of the fur mutant strain especially after 18 h to vancomycin treatment was observed. The treatment of both strains with 150 mg/l polymyxin resulted in normal growth of the wild type and significantly inhibited growth of the fur mutant (Figure 5).

Additionally, two potential ABC transporter systems of the bacitracin/multidrug family (CDIF630erm_00443 – 00445,CDIF630erm_00938 – 00943) and one multi antimicrobial extrusion protein with a downstream MarR family transcriptional regulator gene (CDIF630erm_03501 – 03502) were also found approximately 2- to 3-fold induced at the transcriptional level (Table 1). Multiple genes encoding enzyme of cell wall biosynthesis and modification (*murG*, *murD*, *mraY*, *murF*, CDIF630erm_02905 – 02909, *manC*, *pgm*, *mviN*, *glmU, prs*) were two–fourfold transcriptionally up-regulated under low iron conditions. A mutated *murG* gene was selected to mediate in decreased susceptibility to vancomycin (Leeds et al., 2014). Deletion of the *manC* gene in *Corynebacterium glutamicum* resulted in a slow growing mutant, showing the essential role of the targeted pathway (Mishra et al., 2012). Interestingly, disruption of GDP-mannose synthesis in *Streptomyces coelicolor* resulted in an increased susceptibility to antibiotics of the bacterium (Howlett et al., 2018). The last gene of the operon encoding the transmembrane virulence factor MviN was shown to be essential in *C. difficile* (Chu et al., 2016). Antisense RNA mediated down-regulation of *mviN* resulted in a morphology defects, retarded growth and decreased PSII (integral part of the cell wall anchored glycopolymers) formation and surface deposition (Chu et al., 2016). The bifunctional *N*-acetyltransferase/uridylyltransferase GlmU (CDIF630erm_03829) catalyzes the transfer of an acetyl from acetyl-coenzyme A to glucosamine 1-phosphate to form *N*-acetylglucosamine 1-phosphate during cell wall biosynthesis. The protein is necessary for the infection of various pathogenic bacteria, including *Mycobacterium tuberculosis*, *Yersinia pestis*, *Haemophilus influenzae* and *Xanthomonas oryzae* (Buurman et al., 2011; Min et al., 2012; Patin et al., 2015), it serves as target for the antimicrobial treatment of Mycobacteria (Sharma and Khan, 2017). The *glmU* gene obviously forms an operon with the *prs* gene (CDIF630erm_03828) encoding ribose-phosphate pyrophosphokinase that catalyzes the conversion of ribose-5-phosphate into phosphoribosyl pyrophosphate during nucleotide biosynthesis. The *prs* gene was one of the major up-regulated genes of *Bacillus thuringiensis* in response to erythromycin treatment (Zhou et al., 2018). Both genes were found up-regulated under low iron conditions. The phosphotransferase uptake system for mannose/fructose/sorbose (CDIF630erm_00408 – 00413) was also found enhanced two–threefold at the transcriptional level. Mannose-derived and guanosine-activated compounds are important constituents of the Gram-positive cell wall. In contrast, the genes for the enzymes *N*-acetylglucosamine-6-phosphate deacetylase (NagA) and the *N*-acetylglucosamine-6-phosphate deaminase (NagB) (CDIF630erm_01146 – 01147) involved in cell wall degradation and restructuring were found fourfold down-regulated. The enzyme catalyzes the conversion of *N*-acetyl-D-glucosamine-6-phosphate via D-glucosamine-6-phosphate to D-fructose-6-phosphate during cell recycling. Interestingly, the promoter of the g*lmU* gene is the only gene regulatory element with a potential Fur binding site of almost all iron regulated genes of cell wall metabolism. Obviously, the cell wall is restructured to protect the bacterium against various external challenges. At the same time cell wall degradation and recycling is stopped. Interestingly, the genes for extracytoplasmic function (ECF) sigma factor s^V^ (*csfV*) and the corresponding anti ECF sigma factor RsiV were found enhanced at the transcriptional level. The sigma factor s^V^ regulates peptidoglycan deacetylation and lysozyme resistance (Ho and Ellermeier, 2011; Ho et al., 2014). An iron-regulated gene encoding a peptidyl-prolyl isomerase is encoded by the gene upstream of both genes. The corresponding promoter carried a potential Fur binding site.

### Nucleotide Biosynthesis, CRISPR/Cas System and Prophage Cluster Regulation

Dihydroorotate dehydrogenase (PyrDK), aspartate carbamoyltransferase (PyrB), and orotate phosphoribosyltransferase (PyrE), all enzyme of pyrimidine biosynthesis (*pyrBKDE*, CDIF630erm_00305 – 00308) were found induced at the transcript level under low iron conditions. Interestingly, in other bacteria dihydroorotate dehydrogenase (PyrDK) channels abstracted electrons directly into electron transfer chains and contributes to proton/sodium gradient formation (Reis et al., 2017). Furthermore, the transcripts from an operon (*purECFGNHDL*, CDIF630erm_00340 – 00347) involved in purine biosynthesis were found more abundant (Table 1). The purine GTP serves as precursor of riboflavin biosynthesis, which also was found enhanced under low iron conditions. Both operons revealed Fur binding site containing promoters. The bacterial immunity system against phage infections CRISPR/Cas (CDIF630erm_03259 – 03266) was found approximately twofold down-regulated at the transcriptional and proteomic level (Hargreaves et al., 2014). The prophage encoded by CDIF630erm_01522 – 01532 was also found down-regulated. The corresponding promoter of the operon possessed a conserved Fur binding site.

## Discussion

The highly specialized energy metabolism of *C. difficile* mainly relies on multiple ferredoxin-mediated amino acid utilizing reactions, and on pathways harboring various iron-sulfur cluster containing enzymes (see Figure 3 and Supplementary Table S8). Overall, it is highly iron-dependent. In an anaerobic organism, this usually represents a feasible and effective strategy. There are two major drawbacks of this highly specialized lifestyle. (1) Oxygen is inactivating many of the employed processes. (2) Iron is essential for this type of energy metabolism. We investigated here the critical scenario of low iron conditions. It was no surprise that an initial stress response for the acquisition of iron (iron transporter on!) was observed. Maybe, the production of polyamines has something to do with iron storage and acquisition. But in parallel, a major rebuilding of the central energy metabolism occurred. All ferredoxin-dependent amino acid (Phe, Leu, Gly) utilizing processes were drastically reduced. Flavodoxin as an alternative was brought into the game. Similarly, glucose utilization via pyruvate-formate-lyase, formate dehydrogenase, and hydrogenase, all multi-Fe-S-enzymes, was reduced. Instead, proline utilization directly coupled to the sodium ion/proton pumping RNF complex was strongly enhanced. Thus, the switch from more substrate phosphorylation dominated energy generation to membrane potential based processes obviously required the utilization of a different, membrane potential-dependent ATP-forming ATPase. Most likely, even the membrane composition was adjusted appropriately. Finally, the energy consuming process of motility via flagella movement was reduced. However, the transition period for the adaptation to low iron conditions represents a period of metabolic weakness and physical vulnerability. Here, *C. difficile* “protects the gates,” changing drastically the composition of the cell wall. Protection against antibiotics from other microorganisms of the microbiome, against CAMPs or molecules of the immune system of the host are the major task. And what has Fur to do with all of it? It is the major player, directly and indirectly. Proposed Fur binding sites identified central adaptation processes as directly Fur-controlled. Nevertheless, especially in the complex adaptation of the energy metabolism several indirect regulatory scenarios can be assumed.

In the closely related *C. acetobutylicum* the strong induction flavodoxins and riboflavin biosynthesis under iron limited conditions was also observed besides the expected increase of iron uptake systems (Vasileva et al., 2012). Additionally, a few metabolic enzymes involved in energy generation were found iron controlled, however, not to the degree observed in this study for *C. difficile*. The major difference of *C. difficile* to many other pathogenic bacteria is their aerobic/facultative anaerobic life style. Under these condition iron uptake and storage is connected to ROS formation. Consequently, these bacteria use Fur for the control of superoxide dismutase, catalase, or hydroperoxidase formation (Troxell and Hassan, 2013). Nevertheless, a strict co-regulation of the TCA cycle during virulence by Fur was observed for *Staphylococcus epidermidis* and *Vibrio cholera* (Troxell and Hassan, 2013). Finally, multiple other bacteria employ completely different systems (Irr, RirA, and IscR) for their iron response (Rudolph et al., 2006; Santos et al., 2015; Mandin et al., 2016). Consequently, the observed adaptation of *C. difficile* to low iron conditions partly mediated by Fur is the result of its unique life style and metabolism.

## Author Contributions

AN, MaB, and PD were responsible for the RNA-Seq experiments. CL, KR, SS, SM, DB, and AO performed and interpreted the proteomic approaches. MN-S performed and interpreted the metabolome experiments, bioinformatics came from DE and MiB. MR, MaB and AMM did the electron microscopy studies. DJ, MN-S, MJ, JMBdA and AMM were responsible for data integration and manuscript writing.

## Conflict of Interest Statement

The authors declare that the research was conducted in the absence of any commercial or financial relationships that could be construed as a potential conflict of interest.

## Footnotes

1 http://www.clostron.com/

2 https://github.com/Expression/Analysis/ea-utils

## Supplementary Material

Figure S1 Clostron-based knock out of the *Clostridioides difficile fur* gene. On the left side the mutagenesis strategy and the theoretical PCR-based control with all necessary primers and resulting PCR products are depicted. Corresponding primers and test condition are outlined in the method section. On the right side an experimental PCR-based verification of the generated *C. difficile fur* mutant is shown. The agarose gel with stained DNA from the analysis of wild type versus *fur* mutant clones shows in lane A the wild type DNA control with an expected PCR product of 394 bp. Lane B displays a PCR product of 2,194 bp from the *fur* mutant strain indicating desired mutational insertion, lane C shows the corresponding intron–exon junction PCR product of 410 bp, and lane D displays the spliced ErmRAM marker PCR product of 900 bp. Lane M represents the GeneRuler Ladder Mix, Fermentas (Thermo Fisher Scientific, Darmstadt, Germany).

Figure S2 Growth of wild type and *fur* mutant *C. difficile* in BHI medium. *C. difficile* 630Δerm and corresponding *fur* mutant were grown in BHI medium for 24 h. Growth in anaerobic flasks was monitored every 2 h in at least 3 independent cultivations by measuring the optical density of the culture at 600 nm. The black curve represents 630Δerm growth and the green curve the growth of the corresponding *fur* mutant. Standard deviations are given.

Figure S3 Complementation of the *fur* mutant with *fur in trans*. Growth on high iron containing minimal medium of *C. difficile* wild type (black), the corresponding *fur* mutant (green), and the *fur* mutant complemented *in trans* with the *fur* gene (red) was monitored by absorbance measurements at 600 nm (left, in absorbance units) over the time period indicated (bottom).

Figure S4 Comparison of the growth behavior of *C. difficile* wild type (black) and the corresponding *fur* mutant strain (green) utilizing different iron sources. *C. difficile* 630Δ*erm* and the corresponding *fur* mutant were grown for 24 h *in* CDM medium with different iron sources. The iron sources were: FeSO_4_ **(A)**, Fe citrate **(B)**, hemin **(C)**, FeCl_3_ **(D)**, transferrin **(E)**, and ferritin **(F)**. Growth was monitored spectroscopically every 2 h in anaerobic flasks in at least 3 independent cultivations. Standard deviations are given.

Figure S5 Principle component analysis (PCA) of the RNA-Seq based transcriptome samples from this study in biological triplicates. Cultures were grown in CDM medium under high (760 μg/l) and low (11 μg/l) iron conditions, harvested at mid-log phase (see Figure 1) and used for transcriptome (RNA-Seq) analyses. Obtained results were used for PCA. Orange circles represent wild type grown under high iron, blue circles indicate wild type grown under low iron, orange squares stand for the results for the *fur* mutant grown under high iron conditions, and the blue squares are for the *fur* mutant grown under low iron conditions.

Table S1 Comparative transcriptome (RNA-Seq) analysis of *C. difficile* wild type grown at low and high iron conditions. For details, please consult the Section “Materials and Methods” and “Results”.

Table S2 Comparative transcriptome (RNA-Seq) analysis of *C. difficile* wild type grown at high iron versus the *fur* mutant at high iron conditions. For details, please consult the Section “Materials and Methods” and “Results.”

Table S3 Comparative transcriptome (RNA-Seq) analysis of the *C. difficile fur* mutant grown at low and high iron conditions. For details, please consult the Section “Materials and Methods” and “Results”.

Table S4 Comparative proteome analysis of *C. difficile* wild type and the *fur* mutant grown at low and high iron conditions. For details, please consult the Section “Materials and Methods and “Results.”

Table S5 Genome annotation conversion table for the *C. difficile* 630 (lane A) and *C. difficile* 630Δ*erm* (B). Given is further the gene name (lane C), the start point (D) and end point (E) on the genome, the coding strand (F), the Refseq No. (G), the EC No. of the encoded enzyme (H), the TIGR main role (I), the TIGR minor role (J), the GO terms (K), the name of the gene product (L) and the protein sequence (M).

Table S6 Comparative metabolome and exo-metabolome analysis of *C. difficile* wild type and the *fur* mutant grown at low and high iron conditions. For details, please consult the Section “Materials and Methods” and “Results”.

Table S7 Bioinformatics-based investigation of the Fur-binding sites in the *C. difficile* 630Δ*erm* genome. Listed are all found Fur binding sites found with the consensus shown in Figure 2 upstream from the indicated genes/operons. The results for the Fur binding sites described by Dubois et al. (2016) and from Ho and Ellermeier (2015) are included. For details, please consult the Section “Materials and Methods” and “Results”.

Table S8 Iron-binding proteins in *C. difficile*. Locus tags of *Clostridioides difficile* 630Δ*erm* and their annotation and bound iron as detected by InterPro Scan. Green highlighted locus tags were found regulated in the present experimental set-up (see Table 1).
