# Supplementary figures and images for: Iron Regulation in Clostridioides difficile
Source: Front Microbiol. 2018 Dec 24;9:3183. doi: 10.3389/fmicb.2018.03183 (PMC6311696; doi:10.3389/fmicb.2018.03183)

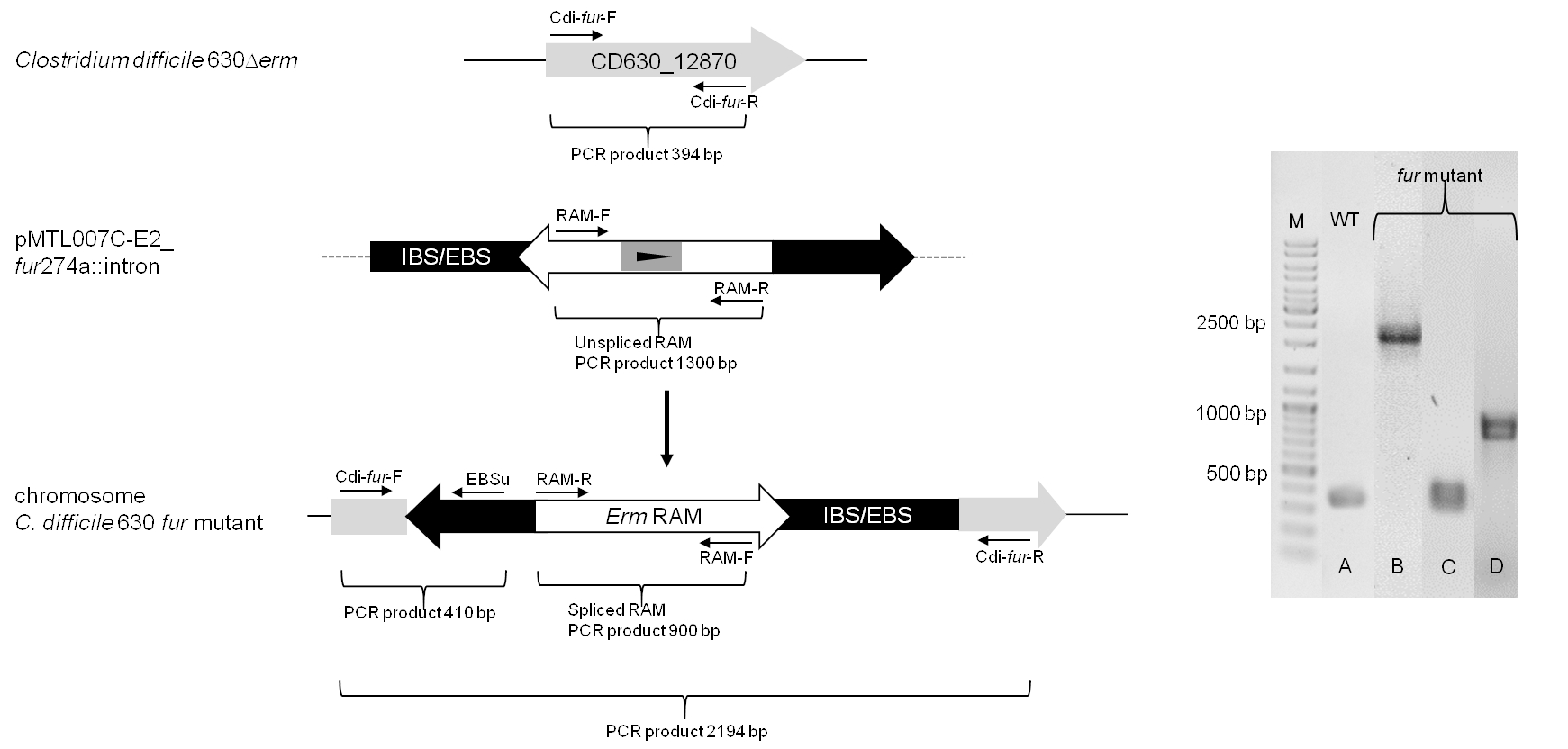

Supplement: Figure S1 — Clostron-based knock out of the Clostridioides difficile fur gene. On the left side the mutagenesis strategy and the theoretical PCR-based control with all necessary primers and resulting PCR products are depicted. Corresponding primers and test condition are outlined in the method section. On the right side an experimental PCR-based verification of the generated C. difficile fur mutant is shown. The agarose gel with stained DNA from the analysis of wild type versus fur mutant clones shows in lane A the wild type DNA control with an expected PCR product of 394 bp. Lane B displays a PCR product of 2,194 bp from the fur mutant strain indicating desired mutational insertion, lane C shows the corresponding intron–exon junction PCR product of 410 bp, and lane D displays the spliced ErmRAM marker PCR product of 900 bp. Lane M represents the GeneRuler Ladder Mix, Fermentas (Thermo Fisher Scientific, Darmstadt, Germany). [file Image_1.TIF]

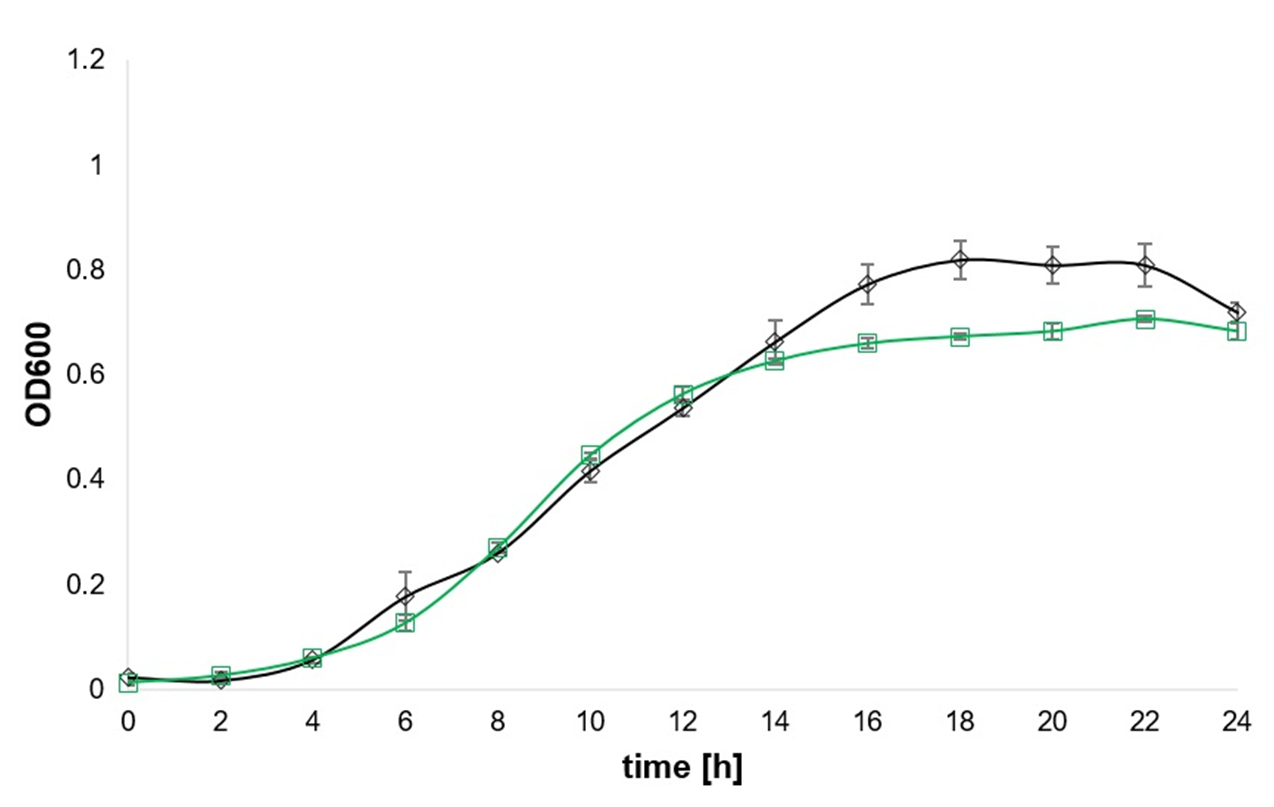

Supplement: Figure S2 — Growth of wild type and fur mutant C. difficile in BHI medium. C. difficile 630Δerm and corresponding fur mutant were grown in BHI medium for 24 h. Growth in anaerobic flasks was monitored every 2 h in at least 3 independent cultivations by measuring the optical density of the culture at 600 nm. The black curve represents 630Δerm growth and the green curve the growth of the corresponding fur mutant. Standard deviations are given. [file Image_2.TIF]

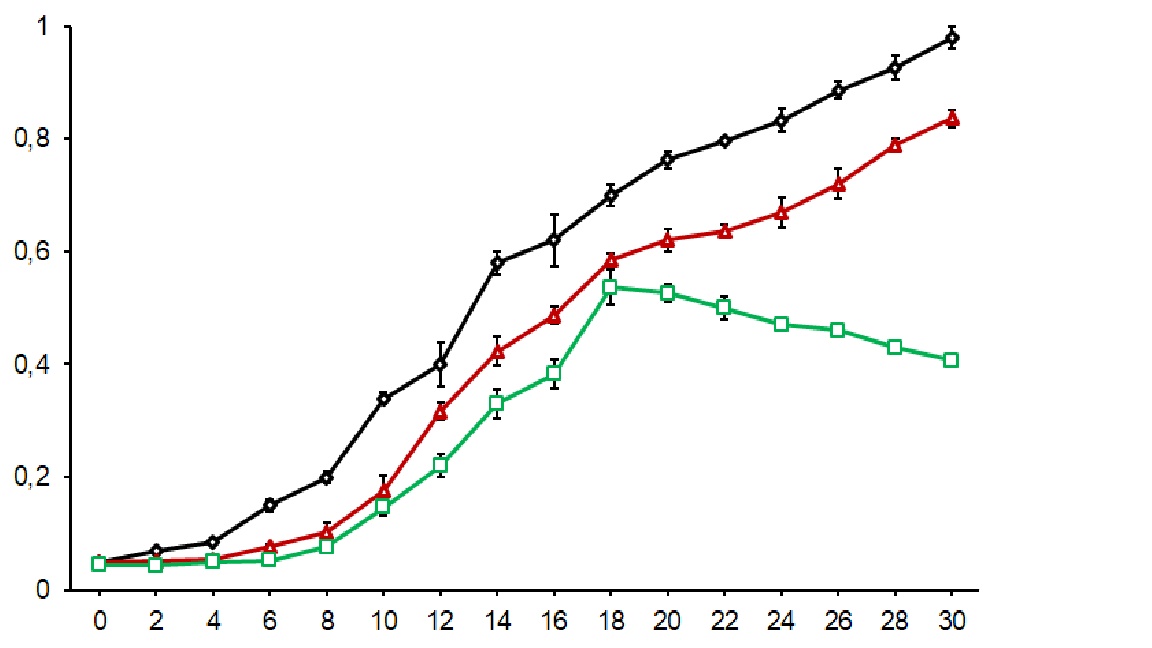

Supplement: Figure S3 — Complementation of the fur mutant with fur in trans. Growth on high iron containing minimal medium of C. difficile wild type (black), the corresponding fur mutant (green), and the fur mutant complemented in trans with the fur gene (red) was monitored by absorbance measurements at 600 nm (left, in absorbance units) over the time period indicated (bottom). [file Image_3.JPEG]

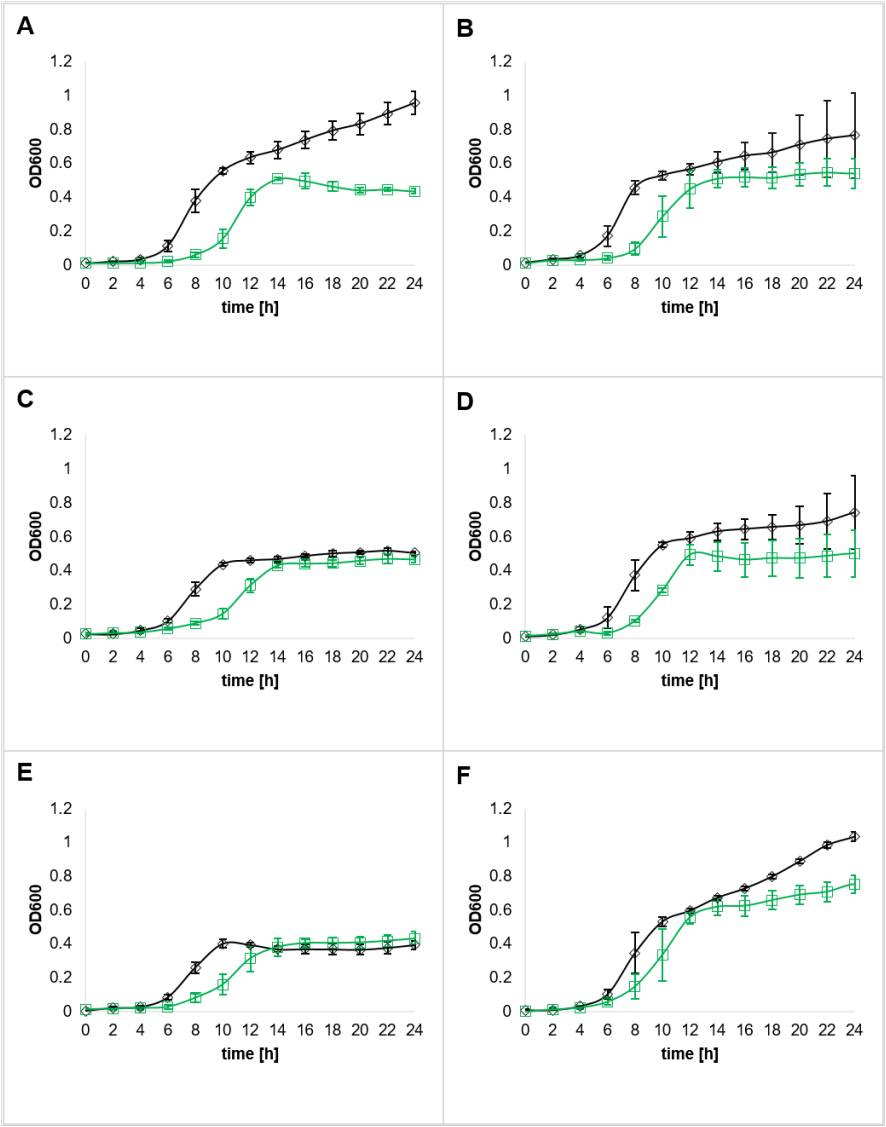

Supplement: Figure S4 — Comparison of the growth behavior of C. difficile wild type (black) and the corresponding fur mutant strain (green) utilizing different iron sources. C. difficile 630Δerm and the corresponding fur mutant were grown for 24 h in CDM medium with different iron sources. The iron sources were: FeSO4 (A), Fe citrate (B), hemin (C), FeCl3 (D), transferrin (E), and ferritin (F). Growth was monitored spectroscopically every 2 h in anaerobic flasks in at least 3 independent cultivations. Standard deviations are given. [file Image_4.TIF]

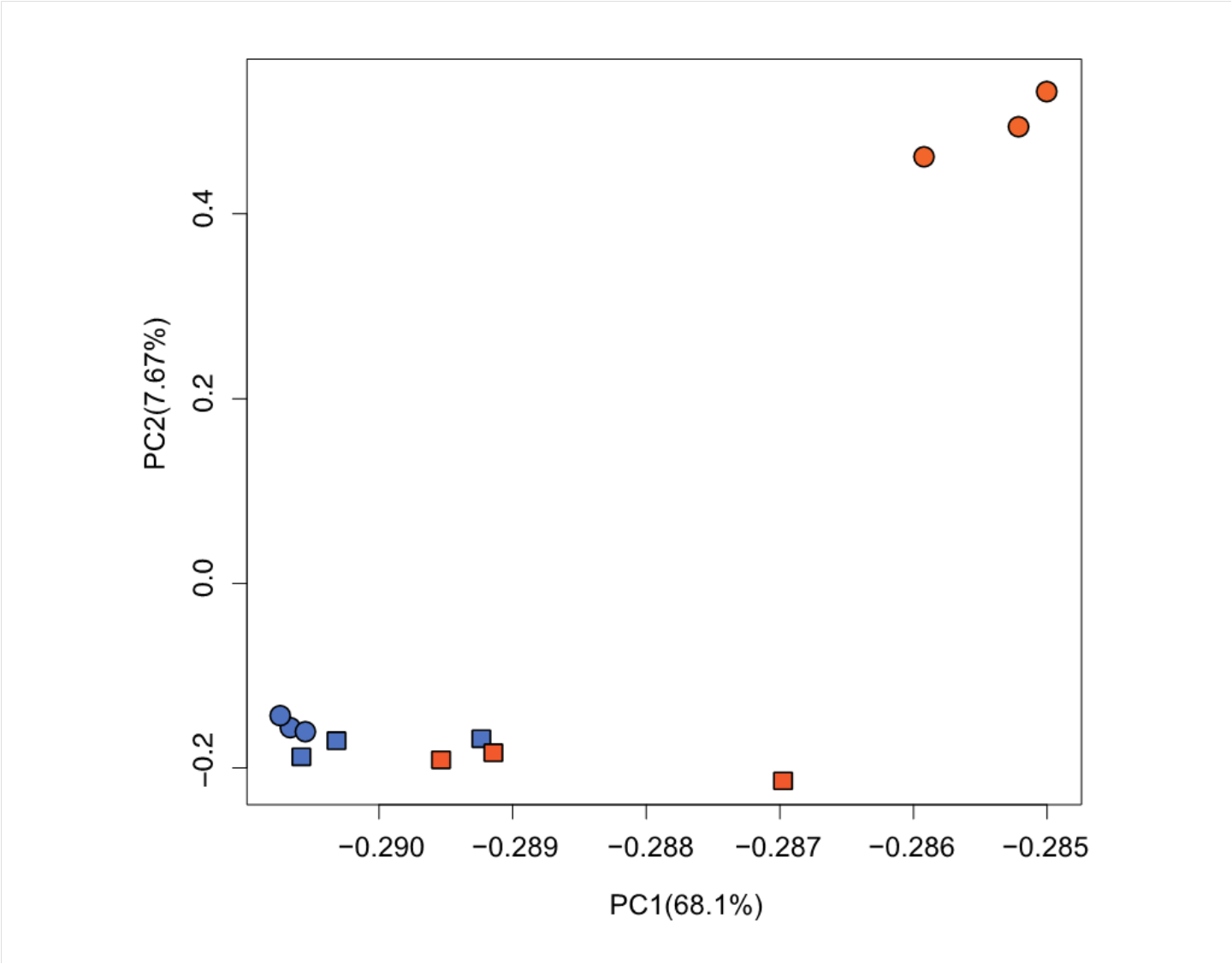

Supplement: Figure S5 — Principle component analysis (PCA) of the RNA-Seq based transcriptome samples from this study in biological triplicates. Cultures were grown in CDM medium under high (760 μg/l) and low (11 μg/l) iron conditions, harvested at mid-log phase (see Figure 1) and used for transcriptome (RNA-Seq) analyses. Obtained results were used for PCA. Orange circles represent wild type grown under high iron, blue circles indicate wild type grown under low iron, orange squares stand for the results for the fur mutant grown under high iron conditions, and the blue squares are for the fur mutant grown under low iron conditions. [file Image_5.TIF]
