# Supplementary material for: Iron Regulation in Clostridioides difficile
Source: Front Microbiol. 2018 Dec 24;9:3183. doi: 10.3389/fmicb.2018.03183 (PMC6311696; doi:10.3389/fmicb.2018.03183)
Supplement: Table S8 — Iron-binding proteins in C. difficile. Locus tags of Clostridioides difficile 630Δerm and their annotation and bound iron as detected by InterPro Scan. Green highlighted locus tags were found regulated in the present experimental set-up (see Table 1). [file Table_8.docx]

**Supplementary table S8. Iron-binding proteins in *C. difficile***. Locus tags of *Clostridioides difficile* 630Δ*erm* and their annotation and bound iron as detected by InterPro Scan. Green highlighted locus tags were found regulated in the present experimental set-up (see Table 1).

| Locus tag | Annotation | Description |
| --- | --- | --- |
| CDIF630erm_00230 | *vorC1* | 4Fe-4S |
| CDIF630erm_00274 | *hpdA* | 4Fe-4S |
| CDIF630erm_00281 | putative radical SAM-family protein | 4Fe-4S |
| CDIF630erm_00294 | *fdxA* | 4Fe-4S |
| CDIF630erm_00297 | *cooF* | 4Fe-4S |
| CDIF630erm_00306 | *pyrK* | 2Fe-2S |
| CDIF630erm_00398 | putative iron-containing alcohol dehydrogenase | Ferrous iron |
| CDIF630erm_00462 | *adhE1* | Ferrous iron |
| CDIF630erm_00525 | *hadB* | 4Fe-4S |
| CDIF630erm_00602 | putative iron-sulfur protein | 4Fe-4S |
| CDIF630erm_00674 | putative aldo / keto reductase | 4Fe-4S |
| CDIF630erm_00676 | *queG* | 4Fe-4S |
| CDIF630erm_00739 | putative ferredoxin | 4Fe-4S |
| CDIF630erm_00740 | putative membrane protein | 4Fe-4S |
| CDIF630erm_00821 | putative nitroreductase-like oxidoreductase | 4Fe-4S |
| CDIF630erm_00846 | ATP-dependent reductive activator of CoFeSP | 2Fe-2S |
| CDIF630erm_00944 | *rbr1* | Ferritin-like |
| CDIF630erm_00946 | *dsr* | desulfoferrodoxin-like |
| CDIF630erm_00952 | *acnB* | 4Fe-4S |
| CDIF630erm_01013 | ferredoxin hydrogenase | 4Fe-4S |
| CDIF630erm_01014 | ferredoxin hydrogenase | 4Fe-4S |
| CDIF630erm_01126 | *leuC* | 4Fe-4S |
| CDIF630erm_01231 | *zupT* | Ferrous Iron |
| \| CDIF630erm_01265 \|  \|  \| \| --- \| --- \| --- \| | *pflC* | 4Fe-4S |
| CDIF630erm_01284 | *rnfC* | 4Fe-4S |
| CDIF630erm_01289 | *rnfB* | 4Fe-4S |
| CDIF630erm_01632 | putative iron-sulfur protein | 4Fe-4S |
| CDIF630erm_01638 | *rbr2* | Ferritin-like |
| CDIF630erm_01641 | *feoA1* | Ferrous iron |
| CDIF630erm_01642 | *feoA2* | Ferrous iron |
| CDIF630erm_01643 | *feoB1* | Ferrous iron |
| CDIF630erm_01673 | putative iron-sulfur binding protein | 4Fe-4S |
| CDIF630erm_01684 | *feoB2* | Ferrous iron |
| CDIF630erm_01685 | *feoA3* | Ferrous iron |
| CDIF630erm_01692 | *rbr3* | Ferritin-like |
| CDIF630erm_01693 | putative iron-sulfur protein | 4Fe-4S |
| CDIF630erm_01704 | *nfnA* | 2Fe-2S |
| CDIF630erm_01769 | ferredoxin | 4Fe-4S |
| CDIF630erm_01808 | *sodA* | Ferrous iron |
| CDIF630erm_01824 | *yclN* | siderophore |
| CDIF630erm_01827 | *yclQ* | Ferrichrome binding |
| CDIF630erm_01878 | putative dinitrogenase iron-molybdenum cofactor | FeMoco |
| CDIF630erm_01901 | *moaA* | 4Fe-4S |
| CDIF630erm_01921 | *mrp* | Fe-S cluster assembly |
| CDIF630erm_01939 | *feoA4* | Ferrous iron |
| CDIF630erm_01956 | putative iron-sulfur protein | 4Fe-4S |
| CDIF630erm_01995 | putative nitrite and sulfite reductase subunit | 4Fe-4S |
| CDIF630erm_02075 | putative iron-sulfur binding protein | 4Fe-4S |
| CDIF630erm_02163 | putative 4Fe-4S binding protein, putative ferredoxin, putative dehydrogenase, putative reductase | 4Fe-4S |
| CDIF630erm_02300 | *preA1* | 4Fe-4S |
| CDIF630erm_02304 | *xdhC1* | 2Fe-2S |
| CDIF630erm_02311 | *xdhC2* | 2Fe-2S |
| CDIF630erm_02323 | *xdhC3* | 2Fe-2S |
| CDIF630erm_02401 | iron-sulfur binding protein | 4Fe-4S |
| CDIF630erm_02410 | putative molybdopterin oxidoreductase, fe-S subunit | 4Fe-4S |
| CDIF630erm_02427 | *ftnA* | Ferritin |
| CDIF630erm_02432 | *vorC2* | 4Fe-4S |
| CDIF630erm_02464 | *asrC* | 4Fe-4S |
| CDIF630erm_02465 | *asrB* | 2Fe-2S |
| CDIF630erm_02466 | *asrA* | 4Fe-4S |
| CDIF630erm_02500 | putative oxidoreductase | 2Fe-2S |
| CDIF630erm_02577 | *gbd* | Ferrous iron |
| CDIF630erm_02621 | *iorA* | 4Fe-4S |
| CDIF630erm_02671 | *vorB3* | 4Fe-4S |
| CDIF630erm_02936 | *pfo* | 4Fe-4S |
| CDIF630erm_03012 | radical SAM maturase | 4Fe-4S |
| CDIF630erm_03112 | *rbr4* | Ferritin-like |
| CDIF630erm_03146 | *fhuD* | Ferrichrome binding |
| CDIF630erm_03283 | Iron family ABC transporter, substrate-binding | Ferric iron |
| CDIF630erm_03250 | *adhE* | Ferrous iron |
| CDIF630erm_03291 | *adhB* | Ferrous iron |
| CDIF630erm_03309 | Putative ferredoxin | 4Fe-4S |
| CDIF630erm_03389 | putative iron-containing alcohol dehydrogenase | Ferrous iron |
| CDIF630erm_03408 | 4fe-4s binding domain protein | 4Fe-4S |
| CDIF630erm_03469 | *xdhA5* | 2Fe-2S |
| CDIF630erm_03471 | *preA2* | 4Fe-4S |
| CDIF630erm_03483 | putative oxidoreductase | 4Fe-4S |
| CDIF630erm_03517 | *sdaB* | 4Fe-4S |
| CDIF630erm_03556 | iron hydrogenase | 4Fe-4S |
| CDIF630erm_03573 | *feoA5* | Ferrous iron |
| CDIF630erm_03574 | *feoB3* | Ferrous iron |
| CDIF630erm_03583 | *pflE* | 4Fe-4S |
| CDIF630erm_03614 | *hydN1* | 4Fe-4S |
| CDIF630erm_03615 | *hydA* | 4Fe-4S |
| CDIF630erm_03616 | *hydN2* | 4Fe-4S |
| CDIF630erm_03619 | *fdhF* | 4Fe-4S |
| CDIF630erm_03711 | hydrogenase (NADP, ferredoxin), ferredoxin-binding subunit | 4Fe-4S |
| CDIF630erm_03712 | hydrogenase (NADP, ferredoxin), iron hydrogenase subunit | 4Fe-4S, 2Fe-2S |
| CDIF630erm_03928 | ferredoxin | 4Fe-4S |
